# The actin nucleator Cobl organises the terminal web of enterocytes

**DOI:** 10.1038/s41598-020-66111-9

**Published:** 2020-07-07

**Authors:** Anne J. Beer, Jule González Delgado, Frank Steiniger, Britta Qualmann, Michael M. Kessels

**Affiliations:** 1 Institute of Biochemistry I, Jena University Hospital - Friedrich Schiller University Jena, 07743 Jena, Germany; 2 Centre of Electron Microscopy, Jena University Hospital - Friedrich Schiller University Jena, 07743 Jena, Germany

**Keywords:** Cell biology, Developmental biology, Molecular biology, Zoology, Gastroenterology

## Abstract

Brush borders of intestinal epithelial cells are mandatory for nutrient uptake. Yet, which actin nucleators are crucial for forming the F-actin bundles supporting microvilli and the actin filaments of the terminal web, in which microvilli are rooted, is unknown. We show that mice lacking the actin nucleator Cobl surprisingly did not display reduced microvilli densities or changes in microvillar F-actin bundles or microvilli diameter but particularly in the duodenum displayed increased microvillar length. Interestingly, Cobl-deficient mice furthermore showed a significant widening of the terminal web. Quantitative analyses of high-resolution cryo-scanning electron microscopy (EM) of deep-etched duodenum samples revealed that Cobl is specifically important for the formation of fine filaments in the central terminal web that connect the apical structure of the terminal web underlying the plasma membrane, the microvilli rootlets and the basal structure of the terminal web with each other. Thus, the actin nucleator Cobl is critically involved in generating one of the cellular structures of the brush border-decorated apical cortex of enterocytes representing the absorptive intestinal surface.

## Introduction

Surface extensions of epithelial cells (enterocytes) by microvilli are mandatory for nutrient uptake in the gut. New-borns with reduced length of microvilli, as it e.g. occurs in the inherited microvillar inclusion disease, thus usually die during their early infancy^1,2^. Yet, despite this importance, it is still unknown which actin nucleators are crucial for forming the F-actin bundles supporting microvilli and the actin filaments of the terminal web, in which microvilli are rooted^3–5^.

Microvilli are uniform membrane protrusions that are structurally supported by a dense bundle of unbranched actin filaments, that are organised in a very dense, almost hexagonal packing by actin-crosslinking proteins and that show very uniform length in brush borders. This length differs along the intestinal tract with about 1300–1400 nm in the small intestine and only 800 nm in the colon^6^. The minus ends of microvillar F-actin bundles reach into the cells as rootlets and are anchored - presumably by plastin 1 (fimbrin) interlinking them with the keratin network^7^ - in the terminal web, which spans the entire apical cortex of enterocytes^8,9^. Although a variety of F-actin-binding proteins including e.g. myosins, tropomyosin, villin, spectrin, ezrin and espin have been identified in biochemical isolations of microvilli and attached terminal web structures^3^, loss-of-function data identifying the actin nucleator(s) that give rise to the F-actin structures in microvilli and/or in the terminal web is lacking. Despite significant scientific efforts during several decades, also microvilli formation and length control in intestinal brush borders still are poorly understood, as i) intestine tissues are not well accessible for molecular manipulations, ii) enterocytes in the murine gut only have a lifespan of a few days and iii) once intestinal microvilli are formed, their shapes are quite uniform during the enterocyte life span. Intestinal microvilli are thus distinct from highly dynamic pseudopodia, filopodia and microvillar structures of other cells but more related to stereocilia – whose F-actin does not turn over within at least several months^10^ and which are found in a variety of sensory cells. In line with this, tip links holding these cellular structures in tight register have not only been found in stereocilia^11^ but recently were also described to interconnect intestinal microvilli^5,12^. In addition, both stereocilia and microvillar F-actin bundles display very high crosslinking of F-actin. Interestingly, even knockout (KO) mice mutually lacking the microvillar actin-bundling proteins plastin 1/fimbrin, villin and espin^13–15^ still developed microvilli - although the microvilli were shortened^16^. Also KO of *Eps8*, which mediates barbed-end-capping at microvillar tips^17^, still yielded microvilli - albeit of reduced length^18^. Tight plasma membrane attachment to the microvillar F-actin bundles in enterocytes is mediated by the ezrin-radixin-moesin (ERM) protein ezrin^5^. However, also enterocytes of *ezrin* KO mice still show microvilli - albeit again with reduced length^19^. As also lack of Arp2/3 complex-mediated actin nucleation did not impair brush border formation^20^, it was exciting that the actin nucleator Cordon-bleu (Cobl)^21,22^ was reported to be required for both formation and growth of microvilli in the human colon adenocarcinoma cell line Ls174T-W4^23^. The observations in Ls174T-W4 cells seemed to be somewhat in line with shorter stereocilia in the sensory lateral line system of *cobl* morpholino-injected zebrafish^24^. However, in contrast to the results of Grega-Larson *et al*.^23^, studies in human choriocarcinoma JEG-3 cells dismissed a requirement of Cobl in intestinal microvillar length establishment, as Cobl deficiency had no effect on microvillar length in these cells^25^.

We generated *Cobl* KO mice^26^ and therefore were able to vigorously address a putative intestinal role of the actin nucleator Cobl directly in the relevant tissue. *Cobl* KO mice showed an increased microvillar length but no lack of intestinal microvilli or changed microvillar F-actin bundles. Importantly, *Cobl* KO mice instead showed a disorganisation of the terminal web and a dramatic loss of interconnecting filaments within the central area of this apical cytoskeletal structure. The discovery of distinct Cobl-dependent filaments in the terminal web provides unique insights into brush border organisation.

## Results

### Cobl is expressed in the murine duodenum, jejunum, ileum and colon and is present in enterocytes

A role of the actin nucleator Cobl^21^ in brush border formation remained unclear due to conflicting observations in two different cell lines^23,25^. We have generated *Cobl* KO mice^26^. We therefore were able to address the role of Cobl directly in the relevant tissue, the three segments of the small intestine and the colon.

**Figure 1:**
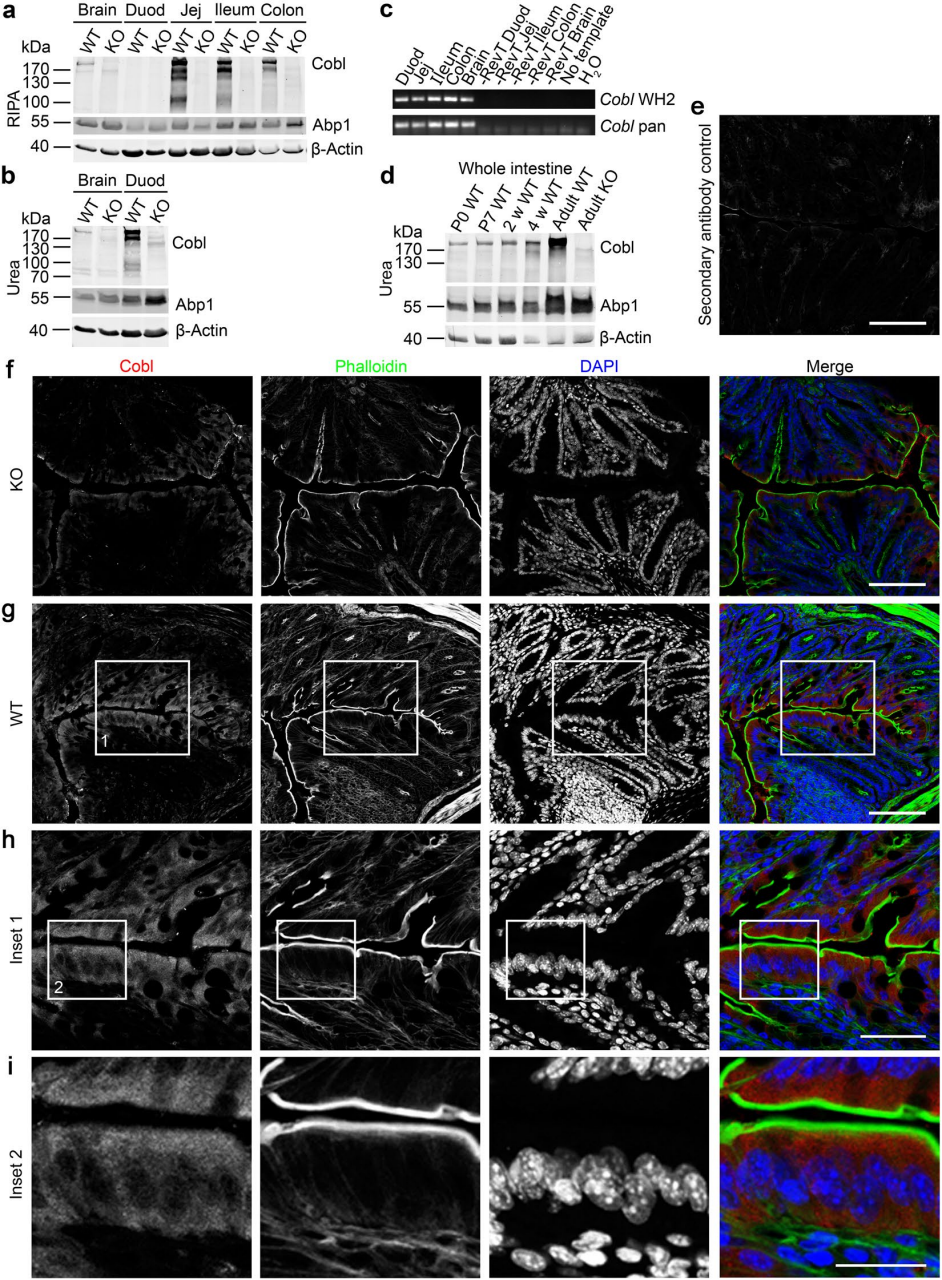
Cobl is expressed in small and large intestine with its highest expression levels at adult age. **(a,b)** Anti-Cobl immunoblotting showing the Cobl expression in duodenum (Duod), jejunum (Jej), ileum and colon as well as in brain by comparing WT and *Cobl* KO tissue extracts of adult mice generated in RIPA buffer **(a)** and in urea-containing lysis buffer **(b)**, respectively. 50 µg protein per lane were loaded. Abp1 and β-actin serve as comparisons. **(c)** RT-PCRs with two different primer pairs for *Cobl* mRNA detection (*Cobl* WH2; *Cobl* pan) in duodenum, jejunum, ileum and colon. Expression in brain was used as reference. Controls are without reverse transcriptase (-RevT), without template (no template) and merely with water (H_2_O), respectively. **(d)** Cobl expression in whole intestinal extracts ranging from P0 to adult mouse tissue. 50 µg protein per lane were loaded. **(e–i)** Confocal image of a secondary antibody control (anti-guinea pig Alexa Fluor568) of the anti-Cobl immunohistochemical staining of murine intestine **(e)** and confocal composite images of longitudinally cut cryo-sections of murine intestine comparing *Cobl* KO **(f)** and WT **(g)** as well as additional magnifications for the WT sample further displaying the endogenous Cobl expression in enterocytes **(h,i)**. Anti-Cobl immunolabelling is shown in red, phalloidin (F-actin) in green and DAPI (DNA) in blue. The positions of areas magnified are labelled with 1 and 2 in the corresponding lower magnification image. Bars, 100 µm **(e–g)**, 50 µm **(h)** and 25 µm **(i)**.

Immunoblotting of lysates from jejunum, ileum and colon all readily showed Cobl expression levels that even exceeded that in brain – previously the tissue with highest Cobl expression^21^. Cobl expression levels were declining along the intestine (Fig. 1a). Immunosignals of Cobl and its degradation products were absent from *Cobl* KO and thus represented a specific Cobl detection in the intestine (Fig. 1a). In the duodenum, the detection of Cobl turned out to be dependent on the homogenisation buffer used; Cobl detection in duodenum required an urea-based protocol (Fig. 1b). This difference did not represent general effects, as both actin and also the cortical F-actin- and Cobl-binding protein Abp1^27^ were detected rather equally in all samples (Fig. 1a,b). The reasons for this unusual extraction and/or detection behaviour of Cobl are currently unknown. As judged from comparisons to Cobl levels in brain, Cobl levels in the duodenum were comparable to those in jejunum or ileum (Fig. 1a,b). RT-PCR analyses confirmed that Cobl mRNA is present in all parts of the intestine (Fig. 1c). Cobl expression in the intestine was still low at birth and during the gestation period. In adult animals, Cobl expression was high (Fig. 1d).

Anti-Cobl immunohistochemistry confirmed that Cobl is present in the intestine (Fig. 1e–i). The observed anti-Cobl immunostaining was specific, as confirmed by both secondary antibody control incubations of WT duodenum as well as by anti-Cobl antibody incubations of *Cobl* KO tissue (Fig. 1e,f). The anti-Cobl immunolabelling occurred predominantly in the enterocyte layer (Fig. 1g,h). Examinations at higher magnifications showed that Cobl was present throughout the enterocytic cytosol and showed some apical enrichment (Fig. 1i).

### *Cobl* KO does not affect microvilli densities but increases microvilli length in the small intestine

Histological examinations showed that *Cobl* KO mice formed an intact intestine and that the duodenum was marked by intact villi (Fig. S1).

**Figure 2:**
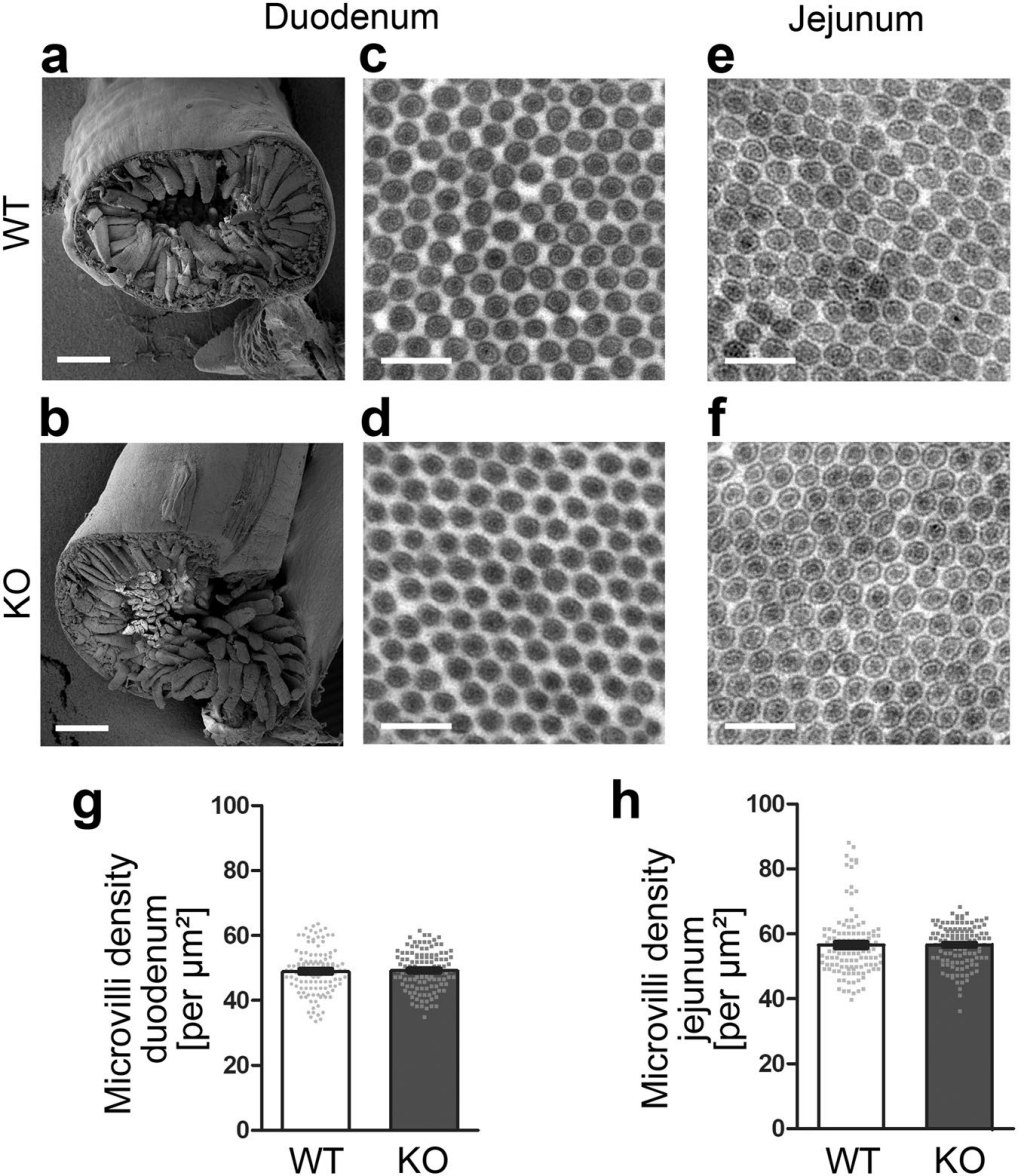
*Cobl* KO mouse duodenum and jejunum show normal microvilli densities. **(a,b)** Scanning EM overview images of cross-sections of duodenum from adult WT **(a)** and *Cobl* KO **(b)** mice. Bars, 400 µm. **(c–f)** Transmission electron micrographs of ultrathin intestinal sections directly comparing WT **(c,e)** and *Cobl* KO **(d,f)** duodenum **(c,d)** and jejunum **(e,f)** tissues. Bars, 400 nm. **(g,h)** Blinded, quantitative analysis of microvilli density from duodenum (**g**) and jejunum (**h**). Data, mean ± SEM (bar/dot plot overlays). n = 120 pictures from 4 mice/genotype and intestinal part each. Statistical analyses, unpaired, two-tailed t-test **(g)** and Mann-Whitney **(h)** (both n.s.).

As light microscopical studies are unable to resolve the number and morphology of microvilli in brush borders, we used EM to closely evaluate whether Cobl is indeed required for microvilli formation. Low resolution scanning EM confirmed that formation of the intestinal epithelium and of its elaborate topology (intestinal villi) was unchanged upon *Cobl* KO (Fig. 2a,b). High-power scanning EM views onto large areas of intestinal epithelia showed dense microvilli-decorations in both genotypes (Fig. S2).

Cross-sections allow for absolutely undoubtable recognition and distinction of individual microvilli. We thus in addition analysed transverse sections across the very regular brush borders of two intestinal segments with high Cobl expression, the duodenum and the jejunum, by transmission EM (TEM) (Fig. 2c–f). Blinded analyses demonstrated that the densities of microvilli in the duodenum and in the jejunum were indistinguishable between WT and *Cobl* KO tissues (Fig. 2g,h).

Cobl overexpression in the human placental epithelial cell line JEG-3 led to a length decrease of F-actin-rich plasma membrane protrusions of microvillar appearance^25^. For the human colon adenocarcinoma cell line Ls174T-W4, in contrast, the length of F-actin-rich membrane protrusions was increased from 2.2 µm to 3.7 µm upon Cobl overexpression and *Cobl* RNAi shortened these cellular structures^23^.

**Figure 3:**
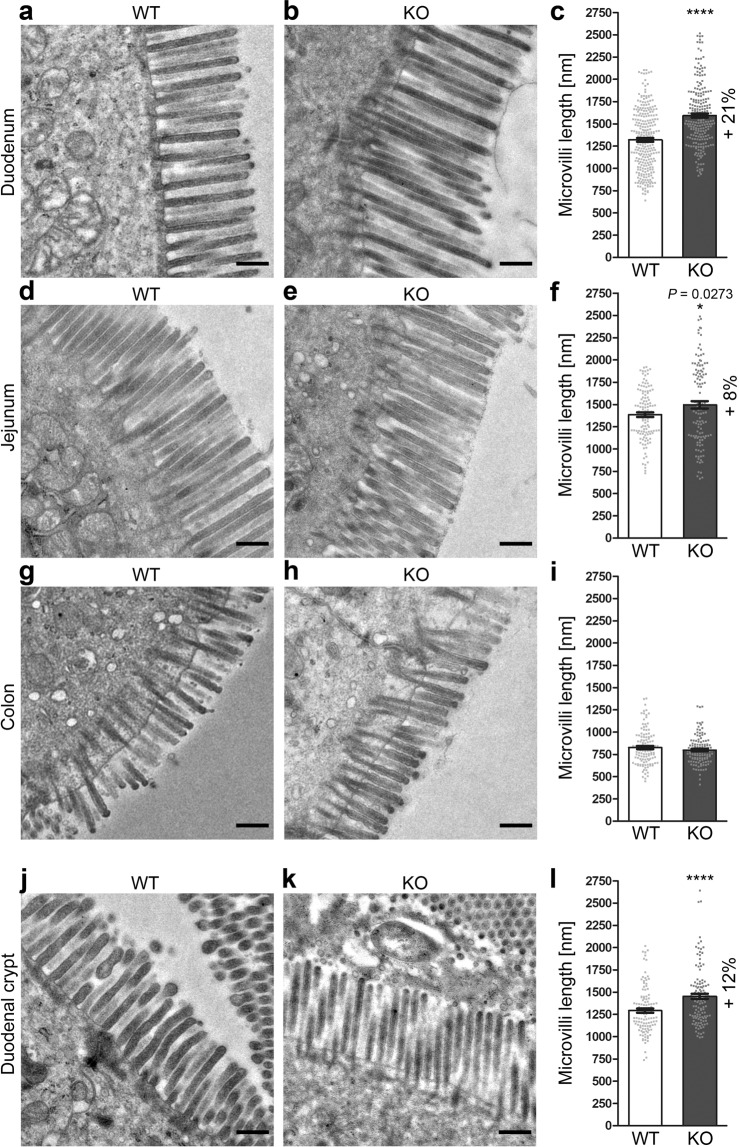
Increased microvilli length in duodenum and jejunum of *Cobl* KO mice. **(a,b,d,e,g,h)** Electron micrographs of ultrathin sections of adult WT **(a,d,g)** and *Cobl* KO mice **(b,e,h)**. Images are from the duodenum **(a,b)**, the jejunum **(d,e)** and the colon **(g,h)**. **(c,f,i)** Blinded, quantitative analysis of microvilli length from duodenum **(c)**, jejunum **(f)** and colon **(i)**. **(j,k)** Electron micrographs of ultrathin sections from duodenum of WT **(j)** and *Cobl* KO mice **(k)** focusing on crypts as places of enterocyte formation and differentiation. **(l)** Blinded, quantitative analysis of microvilli length of enterocytes still in intestinal crypts. Bars, 500 nm. Data, mean ± SEM (bar/dot plot overlays). Jejunum and colon, n = 120 microvilli from 4 mice/genotype each (30 microvilli measured per samples from 1 mouse). Duodenum, n = 270 (WT) and 225 (KO) from 6 (WT) and 5 (KO) mice, respectively (each 45 microvilli measured per samples from 1 mouse). Duodenal crypts, n = 120 from 4 mice/genotype each. Statistical analyses, unpaired, two-tailed t-test **(f)**, Mann-Whitney **(c,i,l)**. *P < 0.05; ****P < 0.0001. For *P* < 0.0001, exact *P* values are not available. Other *P* values (**f**, 0.0273) are presented in the figure.

Sections across intestinal brush borders showed that WT microvilli were 1300–1400 nm long in the small intestine (duodenum and jejunum) and about 800 nm long in the colon (Fig. 3a,c,d,f,g,i). These numbers were well in line with literature for chicken intestine^6^. Strikingly, *Cobl* KO animals showed longer microvilli in both the duodenum and in the jejunum. Upon *Cobl* KO, the deviation from WT microvilli length was +21% in the duodenum (Fig. 3b,c,e,f).

In contrast, in the colon expressing lower levels of Cobl (Fig. 1a), microvilli of WT and *Cobl* KO tissue were identical in length (Fig. 3g–i).

### The microvillar length increase in *Cobl* KO duodenum already occurs in new enterocytes arising from the stem cells in the crypts

During the entire life, new epithelial cells are born from intestinal stem cells, adopt a polar columnar morphology, form microvilli, integrate themselves into the absorptive tissue of the intestine and die a few days later when they reach the tip of intestinal villi^28^. This continuous replenishment of cells occurs in the duodenum in so-called crypts. Enterocytes in crypts thus provide a glimpse at microvilli development^29^. Interestingly, microvilli of the newly generated epithelial cells in crypts of the duodenum already displayed the *Cobl* KO phenotype. Their microvilli already were 12% longer than those of corresponding cells in WT crypts (Fig. 3j–l).

### F-actin organisation in *Cobl* KO microvilli did not differ from WT microvilli in parameters analysed

Besides the length, also the number of actin filaments is a basic parameter in microvillar organisation. The number of actin filaments is reflected by the diameter of the microvillar F-actin bundle, which is in turn reflected by the diameter of the microvillus. High power magnifications of sections of duodenal brush borders clearly showed that *Cobl* KO microvilli had unchanged diameters (Fig. S3a–c). In line, also the diameter of the central F-actin bundle was unaltered (Fig. S3d). Membrane linkage also remained intact, as distortions from the regular, circular microvilli cross-sections were not observed (Fig. S3a,b). Similar results were obtained in the brush border of the jejunum (Fig. S3e–h).

### Cobl accumulates at the base of microvilli and in the terminal web

**Figure 4:**
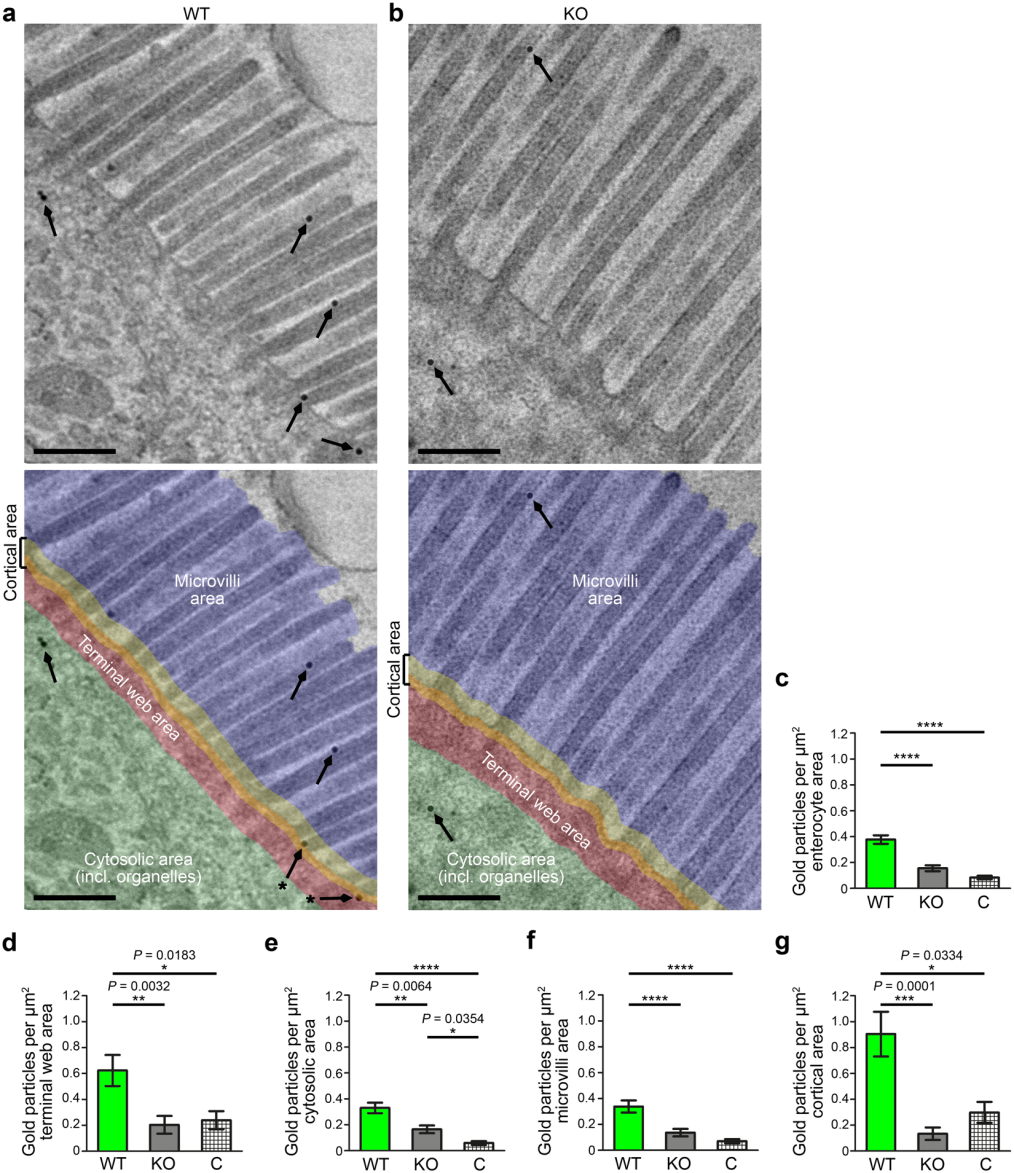
Cobl is primarily located at the cell cortex. (**a,b**) Electron micrographs of anti-Cobl immunogold-labelled ultrathin sections from duodenum of WT (**a**) and *Cobl* KO mice (**b**). Immunogold signals on both WT duodenum sections and background labelling at KO sections are highlighted with arrows. Lower panels with colouring of the microvilli area (transparent blue), of the cytosolic area (transparent green) and of the terminal web area (transparent red) as well as of a cortical area (transparent yellow overlay), which overlaps with both the microvilli base (overlap still appears yellowish) and the apical part of the terminal web (overlap appears orange). For area definitions, please also see the labelling of the coloured panels. Anti-Cobl immunosignals in the cortical area are highlighted with asterisks-marked arrows in the coloured WT panel (**a**; lower panel). (**c–g**) Blinded, quantitative analyses of gold particle density in the following subcellular areas: enterocyte in general (**c**), total terminal web area (**d**), cytosolic areas present in the pictures (**e**), microvilli (**f**) and cortical area (**g**) from WT, *Cobl* KO mice and secondary antibody control (labelled as “C”). Bars, 500 nm. Data, mean ± SEM (for presentations of all individual data points, see dot plot overlaying the bar plots in Fig. S4). n = 48–71 ROIs per area from 2 mice/genotype and condition, respectively. One-Way-ANOVA + Dunn’s multiple comparison (**c–g**). *P < 0.05; **P < 0.01; ***P < 0.001; ****P < 0.0001. For *P* < 0.0001, exact *P* values are not available. Other *P* values are presented in the figure.

We next asked where the actin nucleator Cobl may act in enterocytes and whether filaments other than the densely packed microvillar F-actin bundles may be affected by *Cobl* KO. At the light microscopical level, overexpressed Cobl was reported to enrich in rather cortical regions in different cells including enterocytes^21,23,25,30^. The same was shown in neurons for endogenous Cobl^30^. Transmission EM analyses of anti-Cobl immunogold labelled, ultrathin sections of the duodenum led to relatively frequent and specific anti-Cobl immunogold labelling. The WT anti-Cobl labelling density was significantly above both the labelling density of KO controls and of secondary antibody controls (Fig. 4a–c; Fig. S4; *P* < 0.0001).

The anti-Cobl immunolabelling within the terminal web area showed a particularly high labelling density (Fig. 4d; Fig. S4). Also cytosolic and microvillar areas displayed specific labelling. Yet, the anti-Cobl labelling densities in these areas were roughly merely half as high as in the terminal web (Fig. 4e,f; Fig. S4).

Interestingly, careful distribution analyses of the labelling within both the terminal web area and within the microvilli area revealed that a small subarea overlapping with parts of both of these areas reached the highest anti-Cobl labelling densities (0.9 labels/µm^2^; Fig. 4g; Fig. S4). This Cobl-enriched subarea included the apical terminal web area and the base of the microvilli. It thereby reflected the apical cortex of the enterocytes (“cortical area”; marked by transparent yellow layer on red terminal web and blue microvilli areas in the lower panels of Fig. 4a,b).

### *Cobl* KO leads to structural impairments of the intestinal terminal web

The cytoskeletal mechanisms that organise the terminal web harbouring the microvilli bases are largely unknown. This lack of knowledge is due to the fact that cytoskeletal phenotypes in this important subcellular area are rare and/or were not discovered, yet, as they are technically challenging to address.

**Figure 5:**
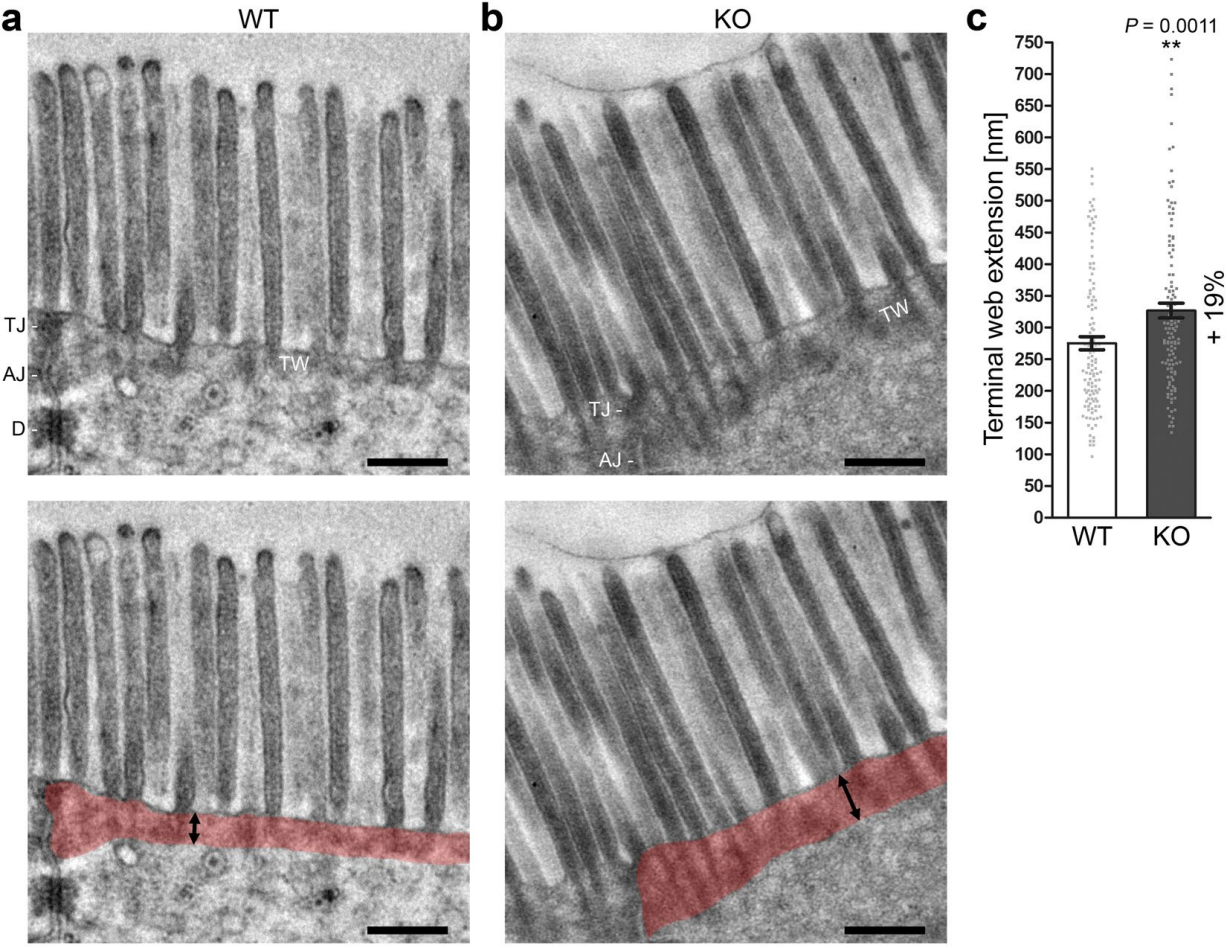
*Cobl* KO duodenal enterocytes have an extended terminal web. **(a,b)** Electron micrographs of ultrathin sections from duodenum of WT **(a)** and *Cobl* KO mouse **(b)**. TW, terminal web; TJ, tight junctions; AJ, adherens junctions; D, desmosome. Lower panels with colouring of the terminal web (transparent red). Double arrows illustrate terminal web thickness measurements. Bars, 500 nm. **(c)** Blinded, quantitative analysis of the extension of the terminal web measured perpendicular to the plasma membrane (examples indicated by black double arrows in **a** and **b**). Data, mean ± SEM (bar/dot plot overlay). n = 120 measurements from 4 mice/genotype. Unpaired, two-tailed t-test. **P < 0.01.

TEM examinations showed that the apical cytoskeleton beneath the duodenal brush borders was altered in *Cobl* KO mice (Fig. 5). The terminal web was visible as largely unstructured electron-dense material with intercalated microvilli rootlets, which were not always clearly resolved. Quantitative analyses showed that the terminal web was disorganised upon *Cobl* KO. It was 19% more extended when compared to WT (Fig. 5a–c).

qPCR analyses showed that the mRNA levels of a variety of other actin filament-promoting factors detected in the gut were not considerably changed upon *Cobl* KO. This included the ancestor of Cobl, Cobl-like, which promotes F-actin formation together with the F-actin-binding protein Abp1^31^, the Arp2/3 complex activators N-WASP^32^ and cortactin^33,34^ as well as the WH2 domain-containing actin nucleator JMY^35^, which was also detected in the intestine (Fig. S5a–c).

Also the mRNA levels of further components that were identified in microvilli/terminal web preparations including myosins^36^ (myosin 2a and myosin 2c were tested), tropomyosin^37^ (tropomyosin 4), plastin 1/fimbrin^14^, villin^13^ and spectrin^38^ (αII-spectrin) all were not considerably changed in *Cobl* KO mice (Fig. S5d–f).

The terminal web phenotype observed in *Cobl* KO mice (Fig. 5) therefore seems to directly reflect a loss-of-function of specifically the actin nucleator Cobl.

### Deep-etching and ultrahigh resolution scanning EM analyses highlight the ultrastructural organisation of the terminal web and the defects caused by KO of the actin nucleator Cobl

The terminal web evaluations by TEM of classical ultrathin section analyses (Fig. 5) raised two technical concerns. First, the cytosolic, basal border of the terminal web often remained somewhat unclear and at times hard to determine in the quantitative analyses. Second, terminal web substructures cannot be addressed, as filament bundles or single filaments in the electron-dense terminal web are not resolved. This severely limited the mechanistic and cell biological insights reachable by conventional TEM. Also high-resolution fluorescence-based methods will be hampered by the very high signal intensities obtained by the detection of the F-actin-rich microvilli rootlets reaching vertically into the terminal web. Ultrastructural analyses of terminal webs of mouse intestine have, to our knowledge, only been reported almost 40 years ago in two seminal studies that subjected scraped off, preextracted cells and cell fragments to a combination of quick-freezing, freeze-fracturing, deep-etching, rotary shadowing and TEM examinations of the resulting platinum/carbon replica^8,9^.

**Figure 6:**
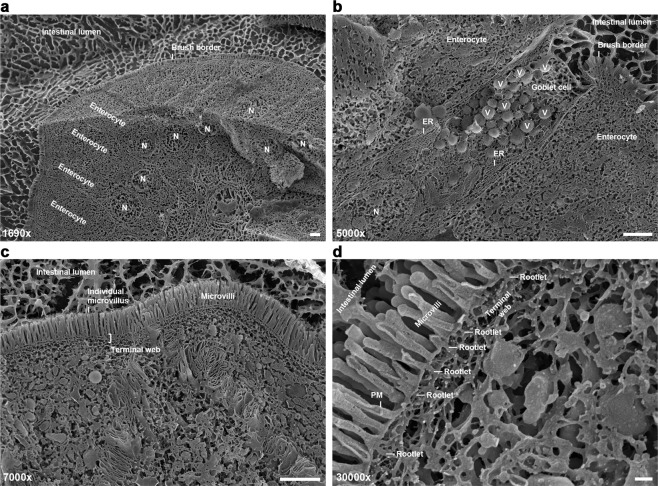
Deep-etching of intestinal samples viewed by cryo-scanning EM is able to provide high-resolution insights into terminal web substructures. (**a**–**d**) Images of mouse duodenum tissue samples that were frozen by liquid nitrogen-cooled propane/ethane, cut, deep-etched at -95 °C for 5 min, sputter-coated with 2–3 nm gold and viewed by cryo-scanning EM at increasing magnifications from 1690fold (**a**), to 5000fold (**b**) and 7000fold (**c**) to 30000fold (**d**). Note that individual enterocytes of the tissue as well as their brush borders and their nuclei (N) can clearly be resolved at 1690fold magnification (**a**), whereas higher magnifications (**b**–**d**) provide more detailed insights into subcellular structures, such as the secretory vesicles (V) of Goblet cells (**b**), the ER (**b**), microvilli, the terminal web in total (**c**) or even allow for visualising structures at the macromolecular level, such as the plasma membrane (PM) and microvillar rootlets within the terminal web (**d**). Bars in **a–c**, 2 µm. Bar in **d**, 200 nm.

We aimed for setting up ultrastructural analyses using intact tissue samples with cells cryopreserved in their native state, as extracting cells for prolonged times will clear them from all dynamic structures. Furthermore, cell rupturing and lack of ATP was reported to lead to an artificial decoration of F-actin structures with myosin fibrils from some unspecifiable intracellular source^8,9^. As TEM of rotary shadowed platinum/carbon replica was not an option for technical reasons, we deep-etched cryopreserved intestinal tissue and directly visualised cellular structures and not platinum replica by 2–3 nm thin gold-sputtering and high-resolution cryo-scanning EM. Overview images showed that such a procedure indeed preserved the intestinal tissue organisation (Fig. 6a). The columnar, epithelial organisation was clearly observable (Fig. 6a). At higher magnifications (Fig. 6b-d), enterocytes displayed clearly recognisable microvilli. Up to a certain depth, even subcellular structures were resolved in 3D. Deep-etching e.g. followed the topology of the almost 1 µm large, mucin-containing secretory vesicles of Goblet cells. The basal border between the terminal web area and cytosol was also clearly observable (Fig. 6b,c).

Scanning attempts at very high magnifications, such as 50000fold to 81000fold, were often hampered by loss of tissue sample stability. In contrast, cryo-scanning EM at 15000fold and 30000fold magnification usually was successful and clearly visualised both biological membranes and cytoskeletal elements, such as microvilli rootlets and further filaments within the F-actin-rich terminal web (Fig. 6d).

**Figure 7:**
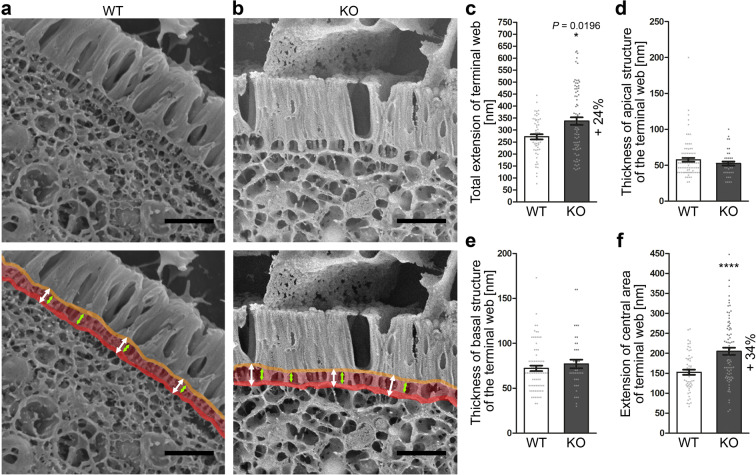
Deep-etching/cryo-scanning EM analyses demonstrate that specifically the central area of the terminal web is widened upon *Cobl* KO. **(a,b)** Cryo-scanning EM images taken from deep-etched duodenum samples of WT **(a)** and *Cobl* KO mice **(b)**. Lower panels represent coloured versions of the shown cryo-scanning EM images with the three parts of the terminal web, the apical structure (plasma membrane and support structures; orange), the central terminal web area (pale red) and the basal structure of the terminal web (red). Arrows visualise the measured extension of the terminal web (white arrows) and of the central area of the terminal web (green arrows). Bars, 500 nm. **(c–f)** Blinded, quantitative analyses of the extension of the terminal web in total **(c)**, of the thickness of the apical structure **(d)**, of the thickness of the basal structure **(e)** and of the extension of the central area of the terminal web **(f)**. Data, mean ± SEM (bar/dot plot overlays). Total terminal web extension **(c)** and extension of the central area of the terminal web **(f)**, n = 56 (WT), n = 78 (KO) measurements on 28 (WT) and 39 (KO) images, respectively, from 2 mice/genotype. Thickness of the apical structure of the terminal web **(d)**, n = 88 (WT), 40 (KO) measurements at 22 (WT) and 10 (KO) high-magnification images. Thickness of the basal structure of the terminal web **(e)**, n = 76 (WT), 40 (KO) measurements at 19 (WT) and 10 (KO) suitable high-magnification images from duodenum samples of 2 mice/genotype. Mann-Whitney test (**c-f**). *P < 0.05; ****P < 0.0001. For *P* < 0.0001, exact *P* values are not available. Other *P* values are presented in the figure.

Fully blinded quantitative analyses of deep-etched duodenum samples showed that the terminal web of *Cobl* KO mice was indeed widened (Fig. 7a–c). In order to unveil, which parts of the terminal web might be affected upon *Cobl* KO, we next addressed the different substructures of the terminal web visualised by deep-etching and cryo-scanning EM. Neither the extension of the plasma membrane-containing apical structure of the terminal web of enterocytes (Fig. 7a,b; orange in coloured panels) nor the basal structure of the terminal web (Fig. 7a,b; red in coloured panels) were altered upon *Cobl* KO (Fig. 7d,e).

This suggested that it was solely the central area of the terminal web that was widened in *Cobl* KO when compared to WT. Independent, blinded measurements of this central area of the terminal web indeed showed a highly statistically significant increase in width by +34% in *Cobl* KO mice (Fig. 7f; *P* < 0.0001).

### KO of the actin nucleator Cobl leads to a loss of filaments interconnecting the different substructures within the central area of the terminal web

In order to address putative structural defects underlying the observed widening of the terminal web in intestinal epithelia of *Cobl* KO mice, we first analysed the microvilli rootlets integrated into the terminal web. In line with the unchanged F-actin bundle diameters inside of microvilli (S3), the rootlet diameter was unchanged upon *Cobl* KO (Fig. S6).

**Figure 8:**
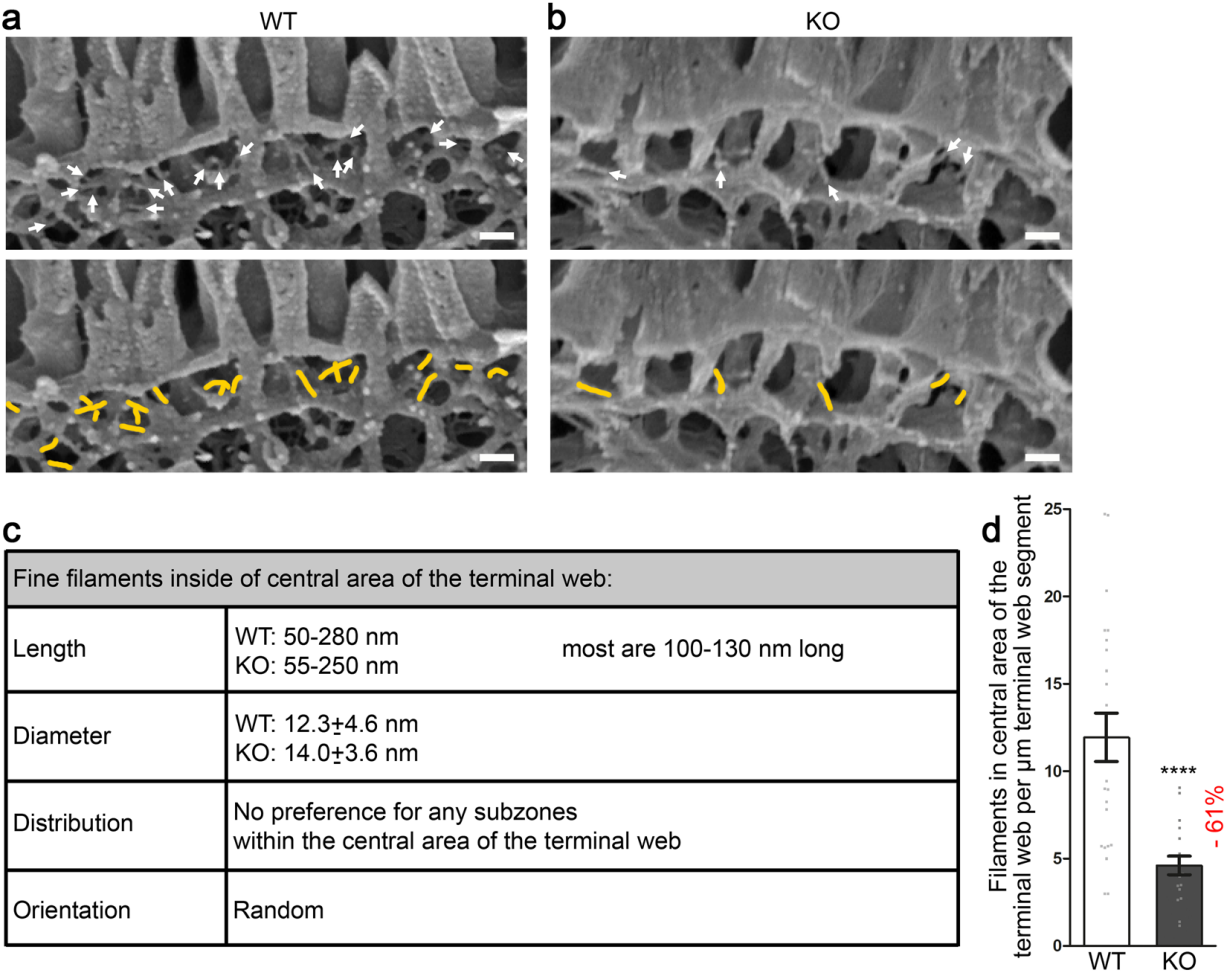
High-resolution cryo-scanning EM of deep-etched tissue samples show that *Cobl* KO mice largely lack fine filaments interconnecting the cell cortex, the rootlets and the basal structure of the terminal web. **(a,b)** High-magnification cryo-scanning EM images of deep-etched duodenum of WT **(a)** and *Cobl* KO mice **(b)**. Note the strong reduction of the fine filaments (arrows) in the central area of the terminal web of *Cobl* KO mice. Lower panels show the images with examples of filaments visualised in yellow. Bars, 100 nm. **(c)** Detailed characterisation of the fine filaments in the central area of the terminal web. Note that the diameters measured include 2times 2-3 nm gold coating. **(d)** Blinded, quantitative analyses of the numbers of the fine filaments in the central area of the terminal webs of 23 WT and 18 KO images from duodenum samples of 2 mice/genotype (expressed as filaments per µm terminal web segment). Data, mean ± SEM (bar/dot plot overlay). Unpaired, two-tailed t-test. ****P < 0.0001. For *P* < 0.0001, exact *P* values are not available.

We next analysed the central terminal web area more carefully. Cryo-scanning EM of our deep-etched samples allowed for 3D visualisations of terminal web substructures up to 200 nm in depth. Visual examinations thus became independent of the random orientations of structures hit by ultrathin sectioning and the subcellular organisation of the terminal web of enterocytes in the small intestine was resolved at nanometre resolution in 3D for a depth of about 200 nm. Besides the apical and the basal structure of the terminal web as well as the microvilli rootlets, another, to our knowledge not yet described, structural element was present in the terminal web of WT mice: Fine filaments that had a relatively uniform diameter of 13 ± 5 nm (including 2–3 nm gold sputtering at each side) (Fig. 8a,b). Their length was highly varying from 50–280 nm in both WT and KO mice (Fig. 8c). Therefore, these filaments cannot represent a single molecule, such as myosin 2 or dimers thereof. Also in contrast to F-actin-associated myosins, the fine filaments neither had a defined orientation in relation to the F-actin bundles in rootlets nor were they restricted to a certain subzone of the central terminal web area. Instead, they were connecting the terminal web subelements, i.e. the rootlets, the apical structure of the terminal web and/or the basal structure of the terminal web (Fig. 8a–c).

Quantitative analyses of blinded high magnification images of intestinal brush borders demonstrated that fewer of these terminal web subelement-interconnecting filaments were present in the central terminal web of *Cobl* KO mice compared to WT mice. More than 61% of the connecting filaments were lacking in *Cobl* KO mice (Fig. 8d).

In line with Cobl’s enrichment in the terminal web of enterocytes, the actin nucleator Cobl thus specifically plays a role in the organisation of the apical cytoskeleton underlying brush borders and the observed *Cobl* KO phenotypes highlight previously unknown principles in the organisation of the terminal web.

## Discussion

Our studies of mice deficient for the actin nucleator Cobl demonstrated that specifically previously unidentified, fine filaments that are interconnecting the apical structure of the terminal web, the microvillar rootlets and the basal structure of the terminal web and that are thus located in the central area of the terminal web of enterocytes were affected by *Cobl* KO. These defects were accompanied by impairments in microvilli length control and disturbances of terminal web organisation – two rarely observed phenotypes providing previously unreachable, detailed insights into the organisational elements of intestinal brush borders.

Yet, our findings are in clear contrast to a recent study, which suggested that *Cobl* knock-down causes impairments in microvilli formation^23^. The apparent discrepancy likely reflects that the Cobl-dependent cortical F-actin-rich structures, which Grega-Larson *et al*.^23^ described as “brush borders” in Ls174T-W4 cells, rather represented dynamic filopodia, pseudovilli or other dynamic cortical F-actin-rich structures but no bona fide microvilli. In line, the protrusions observed in Ls174T-W4 cells were much longer (2.2 µm) than the tightly controlled physiological length of microvilli, and were observed in cells that were neither confluent nor of epithelia-like, columnar morphology but were acutely cell polarity-induced^23^. Also, the F-actin-rich structures affected by *Cobl* RNAi were not well-resolved, as quantitative scoring was done using merely low resolution light microscopy (40x objective^23^). In contrast, our analyses were directly conducted in the highly organised brush borders of the intestine, with *Cobl* loss-of-function brought about by *Cobl* KO^26^ and at 7000–40000fold magnifications allowing for detailed analyses of microvillar parameters. Microvilli densities were identical in both the duodenum and the jejunum of WT and *Cobl* KO mice. The *Cobl* KO mice analysed represent a ubiquitous and constitutive *Cobl* KO generated by Cre/lox exon 11 targeting eradicating specifically all actin nucleating products of the *Cobl* gene^26^. Nevertheless, microvilli were present in *Cobl* KO mice without any significant changes in microvilli density in both the duodenum and the jejunum. We thus firmly conclude that the actin nucleator Cobl does not play a critical role in this cell biological process. The unchanged densities of microvilli in both *Cobl* KO duodenum and jejunum tissue samples are in line with the fact that *Cobl* KO mice did not show any dysfunctions of intestinal absorption, which would manifest in malnutrition, diarrhoea and/or death. Instead, *Cobl* KO mice develop relatively normal and show no deviations in body weight^26^.

We observed that both in the duodenum and in the jejunum, *Cobl* KO did not cause any lack in microvilli but led to an increase of the length of microvilli. Microvillar length increases were thus far rarely observed in KO mice. To our knowledge, only the intestine of *Vitamin D Receptor* KO mice^39^ and oocytes of mice deficient for the Ca^2+^-activated Cl^−^ channel Ano^40^ show such defects. In both cases, these defects are not fully understood at the molecular level but may indirectly be mediated by some ERM protein expression changes^39,40^.

The microvilli length increase in *Cobl* KO mice may reflect slight changes in actin dynamics. Considering the relatively slow turnover rates of microvillar F-actin bundles, these deviations may be very subtle but over time may eventually lead to some length increases. Possible, mutually not exclusive mechanisms include an increase of the availability of free G-actin caused by the lack of the actin nucleator Cobl and by the resulting lack of the actin filaments Cobl would form in WT cells. The pool of polymerisation-competent actin monomers is limited. Thus, the differently formed F-actin structures of a cell would compete with each other for actin monomers. Such internetwork competitions can explain the growth of one cytoskeletal structure at the expense of another^41^. As Cobl associates and/or stays associated with F-actin^21^, it is theoretically also possible that in the absence of Cobl, F-actin binding sites become available, which are then taken over by F-actin binding proteins. This may then cause subtle changes in F-actin dynamics. It is unclear, whether the relatively moderate density of Cobl we observed along the F-actin bundles in WT microvilli would be sufficient to mediate the latter scenario but microvillar length increases have e.g. been detected upon espin overexpression^42^.

Instead of a role in microvilli formation, we demonstrated that Cobl is crucial for proper terminal web formation. These results are in line with the relatively high anti-Cobl immunogold labelling density we determined in particular at the apical cortex. In general, the discovered role of the actin nucleator Cobl at the cell cortex of enterocytes is in line with other observations of Cobl-mediated F-actin formation in the proximity of the cortex of different cells, such as Cobl’s ability to i) induce three-dimensional, F-actin-rich membrane ruffles when overexpressed in COS-7 cells^21^ and to ii) bring about dendritic branches originating from transiently F-actin-rich sites in neurons^21,43^. At the molecular level, some role of Cobl at the cell cortex is well in line with Cobl’s interaction capabilities. First, Cobl interacts with membranes directly^43^. Second, Cobl interacts with Abp1^27^, an F-actin-binding protein accumulating in areas of cortical F-actin formation^44^. Finally, Cobl interacts with membrane-associating BAR domain proteins, such as syndapins^30^, as well as SNX9 and ASAP1/2^25^. Cobl interactions with syndapins (also called PACSINs) are Ca^2+^/CaM-regulated^43^ and crucial for plasma membrane recruitment of Cobl^30^. The requirement of syndapins for proper membrane association of Cobl first shown for neurons^30^ was recently also corroborated for enterocytes^45^.

Our ultrastructural analyses demonstrated that *Cobl* KO led to a widened terminal web of enterocytes and to a lack of filaments that connect the cell cortex, the microvilli rootlets and/or the basal structure of the terminal web with each other. To our knowledge, a disorganisation of the terminal web has only been observed in one other KO mouse thus far: Also *ezrin* KO led to a widening of the terminal web. However, in the case of *ezrin* KO, this defect of the terminal web was accompanied by shortened microvilli^19^ and not by a length increase, as we observed upon *Cobl* KO. Thus, microvillar length increases and impairments in the organisation of the terminal web are mechanistically independent KO phenotypes.

Careful analyses of the terminal web are hampered by technical limitations, as standard methods only allow for rather superficial examinations. Our analyses of the brush border of duodenal enterocytes at the ultrastructural level clearly demonstrated a critical role of Cobl in the organisation of the terminal web. To our knowledge, substructures of the terminal web have never been quantitatively examined in any KO mice at the ultrastructural level. Our combination of deep-etching and cryo-scanning EM provided detailed ultrastructural information. We were able to demonstrate that the thickness of the apical structure of the terminal web, of the rootlets and of the basal structure of the terminal web were not affected. Instead, specifically fine filaments of a relatively uniform net thickness of 7 nm (i.e. without the gold sputtering) connecting the apical structure of the terminal web with the rootlets and/or the basal structure of the terminal web showed a strongly reduced abundance in the central terminal web of *Cobl* KO mice. It is not possible to unambiguously prove by the methods used that these structures lacking upon KO of the actin nucleator Cobl are actin filaments. However, their thickness of exactly 7 nm and their very strongly varying length is a strong hint towards F-actin.

In contrast, attribution to single molecules, such as myosins, seems unlikely, as e.g. coiled coil structures as in myosin tails only have a diameter of little more than two α-helices (1.2 nm) and thus would require higher magnifications for detection than we can technically reach. Single molecules would also show very uniform length. Consistently, superfine filaments that seemed to be myosins had been observed between rootlets when platinum/carbon replica of extracted, deep-etched epithelia had been viewed at 121000x to 144000x magnification. Their length seemed quite uniform and suitable for interlinking rootlets. Interestingly, they could only be detected when extracted epithelia or cell fragments were used, i.e. cells were depleted for ATP^8,9^.

We aimed for setting up ultrastructural analyses based on scanning EM using intact tissue samples with cells cryopreserved in their native state. The Cobl-dependent filaments in the terminal web of the duodenum thus do not depend on artificial energy depletion by prolonged extraction procedures or analyses of cell fragments. Furthermore, the Cobl-dependent filaments we identified showed highly varying lengths and various orientations in the terminal web and did not just interlink rootlets by relatively uniform bridges. This argues that they are not composed of a certain molecule but are polymers with varying modes of interactions. The Cobl-dependent filaments we observed were found to be anchored to rootlets, to the plasma membrane and/or the apical structure of the terminal web and to the basal structure of the terminal web, respectively, in various orientations.

A very specific responsibility of the actin nucleator Cobl for a distinct subset of filaments has also been observed in the cuticular plate beneath the sensory apparatus of outer hair cells of the cochlea^26^. No ultrastructural data are available for actin cytoskeletal elements inside outer hair cells of *Cobl* KO mice. Yet, it is attractive to speculate that the specific loss of a certain subfraction of actin filaments beneath these ciliary and stereociliary arrays may be mechanistically related to the observed loss of connecting filaments in the central area of the terminal web of enterocytes in *Cobl* KO intestines.

Taken together, quantitative analyses using both classical ultrastructural methods and a combination of deep-etching and high-resolution scanning EM of intact intestinal tissue samples provided detailed structural insights into the largely unknown cell biology of the terminal web. Our analyses revealed that the actin nucleator Cobl plays an important role in organising the apical cytoskeleton underlying intestinal microvilli by being critical especially for a subset of filaments interconnecting the different structural elements of the terminal web.

## Methods

### *Cobl* KO mice

Mice lacking the actin nucleator *Cobl* KO mice were generated as described^26^. The mice were kept in a C57BL/6J::129/SvJ (99.7::0.3) genetic background, were bred heterozygously and were housed under 14 h light/10 h dark conditions with *ad libitum* access to food and water.

Animal procedures were performed in strict compliance with the EU guidelines for animal experiments and were approved by the local government (permission number of breeding permission: UKJ-17-021; Thüringer Landesamt für Verbraucherschutz, Bad Langensalza, Germany).

Mice were sacrificed by cervical dislocation or CO_2_ inhalation to obtain tissue material for examinations.

### Antibodies and reagents

For Western blot analysis, guinea pig anti-Cobl^DBY^^30^ and guinea pig anti-Abp1 antibodies^44^ were affinity-purified according to procedures described before^46^. Mouse anti-β-actin antibodies were from Sigma-Aldrich.

Corresponding secondary antibodies used were donkey anti-guinea pig IRDye800, donkey anti-guinea pig IRDye680 (LI-COR Bioscience) and Alexa Fluor680-labelled goat anti-mouse (Thermo Fisher Scientific Inc.).

For immunohistochemistry, Cobl^DBY^ guinea pig serum was applied and detected by Alexa Fluor568-labelled goat anti-guinea pig antibodies (Thermo Fisher Scientific Inc.). F-actin was detected with Alexa Fluor488-coupled phalloidin (Thermo Fisher Scientific Inc.).

The guinea pig anti-Cobl^DBY^ antibodies were additionally used in immunolabelling of ultrathin sections and detected by 15 nm gold-coupled goat anti-guinea pig antibodies (British Biocell International Ltd.).

### Western blot analysis

Mice brains were collected and immediately snap-frozen in liquid nitrogen. Guts were either taken as a complete organ or dissected into duodenum, jejunum, ileum and colon, washed with PBS and snap-frozen in liquid nitrogen.

Frozen tissues were homogenised either in ice-cooled radioimmunoprecipitation assay (RIPA) buffer containing 50 mM Tris/HCl (pH 8.0), 150 mM NaCl, 1% (v/v) IGEPAL CA-630 (Sigma-Aldrich), 0.5% (w/v) deoxycholate, 0.1% (w/v) SDS, 1x protease inhibitor cocktail (Complete EDTA-free tablets, Sigma-Aldrich) supplemented with 1.5 mM EDTA, 1.5 mM EGTA and 1% (v/v) PMSF (Serva) or in an urea buffer containing 12.5 mM Tris/HCl (pH 8.0), 25 mM KCl, 1.5 mM EDTA, 7 M urea, 2 M thiourea, 70 mM DTT and 2x protease inhibitor cocktail using a Potter S homogeniser (Sartorius).

Samples were cleared from cell debris by centrifugation at 10000xg for 20 min at 4 °C. The supernatants were collected and either immediately snap-frozen or supplemented with SDS sample buffer, boiled for 5 min and subjected to immunoblotting onto nitrocellulose membranes. Equal amounts of protein (50 µg) were loaded.

Detection was performed using a LI-COR Odyssey detection system (LI-COR Bioscience).

### Immunohistochemistry

WT and *Cobl* KO mice were sacrificed by CO_2_ inhalation and perfused transcardially with PBS and 4% (w/v) paraformaldehyde (PFA). Intestinal samples were post-fixed overnight in 4% PFA at 4 °C and afterwards transferred into 10% (w/v) and 30% (w/v) sucrose solution for 24 h each. 8 µm thin cryosections were prepared using a cryostat (CM3050S Leica Biosystems).

WT and *Cobl* KO tissue sections were permeabilised with 0.2% (v/v) Triton X-100 in PBS for 10 min, washed with PBS and blocked in 10% (v/v) fetal calf serum/1% (w/v) BSA in PBS (blocking solution) for 1 h. Primary antibody incubation took place overnight at 4 °C. After washing steps in blocking solution containing 0.2% (v/v) Triton X-100 and in PBS, the sections were incubated with secondary antibodies and with Alexa Fluor488-coupled phalloidin in blocking solution containing 0.2% (v/v) Triton X-100. Subsequently, the sections were washed, stained with DAPI and embedded in Fluoromount-G (SouthernBiotech).

Secondary antibody controls were done in parallel and prepared accordingly, except that blocking solution was used instead of the primary antibody solution.

### Confocal imaging and image procession

Confocal imaging was performed using a TCS SP5 confocal microscope (Leica) equipped with a HCX APO U-V-I 40.0 × 0.75 DRY UV objective and LAS AF software. The microscope was operated under identical settings to obtain quantitatively comparable images.

Image processing was done by ImageJ or Adobe Photoshop. An equal number of z-stacks was processed to maximum intensity projections (MIP). Image processing was performed equally for *Cobl* KO and WT stainings and did not include any gamma adjustments.

### Reverse transcription PCR

RNA isolation and reverse transcription PCR (RT-PCR) were performed as described^27^. In brief, tissue samples from whole brain and intestine were dissected, snap-frozen and homogenised by grinding in liquid nitrogen. Subsequently, samples were resuspended in Trizol reagent (Thermo Fisher Scientific Inc., Invitrogen), treated with DNase (RNase-free DNase kit, Qiagen) and reversely transcribed using oligo(dT)-primers and RevertAid H Minus Reverse Transcriptase (Thermo Fisher Scientific Inc., Fermentas).

In order to test for the absence of contaminating genomic DNA, controls without reverse transcriptase were run in parallel. Expression was determined by gene-specific exon-spanning primers (Table 1) and analysed using *β-actin* or *GAPDH* as control.

**Table 1:** Primers used for RT-PCR and qRT-PCR analyses, respectively.

Gene	Forward primer (5′ → 3′)	Reverse primer (5′ → 3′)
*Abp1*	ACCGACTGGGCTCTTTTTACC	TCACCTTCCCGCTGTTGAG
*β-Actin*	CCCCTGAACCCTAAGGCCA	CGGAGTCCATCACAATGCCT
*Cobl* pan	GGCTCCTGAGAAATCTGTACG	CTAAACATTTCTCTTCTGTTGTCC
*Cobl* WH2	GCTCCGGAAGACTGCAGAACA	CGAGCAAGGGAACCTTTCTTAGTC
*Cobl-like*	TTGCTCACTGCAATCCGGTC	CCCGACTTCCCATTCACAGA
*Cortactin*	ATTGAGGCCGTAACCAGCAA	CTCTCTCGGCTTCTGCCTTC
*GAPDH*	ATTGACCTCAACTACATGGTCTACA	CCAGTAGACTCCACGACATACTC
*JMY*	AGGAAAGAAAAAGTGCCTGGGT	TGAATGCGCCACTCGTAATCT﻿
*Myosin 2a*	GAGCGAGGAGAAGAAGAAGCA	TTGGCTGTGTCAATGTGTGC
*Myosin 2c*	ACAGAGACGAGATGGCAGAGG	CTTTCGGTAATGGTCCTTGAGC
*N-WASP*	CCCCGCAAGAGAACGAGTC	TAACCAGACAAGCGACACCA
*Plastin 1/Fimbrin*	AAGGCGACCGAGATGATGG	AGCCAAGTTTATCCGCTTCCT
*aII-Spectrin (Sptan1)*	CAGGAGGTGTATGGTGCGAT	CTTGATGGAGTTGAAGGTAGCC
*Tropomyosin 4*	GAAATCACTGGAGGCTGCTT	GCCCACATTCTCTTCTTTGGC
*Villin*	TGCTGACGAGGTTATGAGCC	AGTCTTCGGTGGACAGGTG

### Quantitative RT-PCR

Template DNA, i.e. cDNA, from WT and *Cobl* KO tissue was obtained as for RT-PCR. Quantitative RT-PCR (qPCR) was performed with Maxima SYBR Green/Fluorescein qPCR Master Mix (2×) (Thermo Fisher Scientific Inc.) and specific mouse primers (Table 1) at a final concentration of 400 nM each. Amplification was performed using a MyiQ Single-Colour (Bio-Rad Laboratories, Inc.) and a StepOnePlus Real-Time PCR Detection System (Applied Biosystems), respectively, applying the following cycle conditions: 10 min polymerase activation was followed by 30 to 40 amplification cycles depending on the expression level. Amplification cycles were performed with 95 °C for 20 s, 60 °C for 40 s and 72 °C for 1 min (MyiQ Single-Colour) and 95 °C for 15 s, 60 °C for 30 s and 72 °C for 30 s (StepOnePlus), respectively. Melting curve analysis consisted of 8 s (MyiQ Single-Colour) and 10 s (StepOnePlus), respectively, measuring temperature according to the predetermined melting peak, 1 min at 95 °C and 1 min at 55 °C.

Data were processed by MyiQ Optical System Software 1.0 (Bio-Rad Laboratories, Inc.) and StepOnePlus Software (Applied Bioscience), respectively. Fold changes comparing WT and *Cobl* KO were calculated using ∆∆Ct with normalisation to either *β-actin* or *GAPDH*.

### Scanning EM analyses of epithelial surfaces of intestinal tissue samples

Heart perfusions were performed using PBS and subsequently 4% (w/v) PFA. Pieces of mouse intestine (approx. 3 mm edge lengths) were fixed with 2.5% (v/v) glutaraldehyde and 4% (w/v) PFA in 0.1 M sodium cacodylate buffer pH 7.2 for 2 h. The samples were washed three times with 0.1 M sodium cacodylate buffer and were then dehydrated with increasing ethanol concentrations (30, 50, 70, 80, 90 and 100% (v/v)).

Afterwards, samples were dried with a CPC 030 critical point dryer (BAL-TEC) using liquid CO_2_ and coated with gold (2–3 nm coating thickness) by a SCD005 Sputter Coater (BAL-TEC).

Images were taken with a Leo 1530 Gemini scanning electron microscope (Carl Zeiss AG), recorded digitally with a secondary electron detector at 8 kV and processed by ImageJ and Adobe Photoshop software.

### Ultrathin sectioning and TEM

Mice were sacrificed and transcardially perfused using first PBS and subsequently 4% (w/v) PFA. The gut was dissected and mouse intestine samples with a size of approx. 1 mm^3^ were incubated in 0.1 M sodium cacodylate buffer (pH 7.2), 2.5% (v/v) glutaraldehyde, 4% (w/v) PFA for 2 h and washed thrice with 0.1 M sodium cacodylate buffer. Increases in contrast were achieved by incubation with 1% (w/v) OsO_4_ for 1 h. After washing with 0.1 M sodium cacodylate buffer, samples were dehydrated by increasing ethanol concentrations (30, 50, 70, 80, 90 and 100% (v/v)) and stained with uranylacetate (2% (w/v) uranylacetate in 50% (v/v) ethanol). The samples were then embedded in araldite resin for 48 h at 60 °C.

Ultrathin sectioning of embedded samples was performed using a LKB 8800 A Ultratome III (LKB Produkter AB). 60 nm thin sections were collected onto formvar-coated grids and stained with lead citrate solution (Electron Microscopy Sciences) for 2 min.

Samples were blinded and images were taken using an EM902A transmission electron microscope (Carl Zeiss AG) operated at 80 keV. Images were recorded digitally using a 1 k FastScan CCD camera (TVIPS camera and software).

### Quantitative evaluations of TEM images of intestinal brush borders

All analyses were conducted using blinded TEM images.

For evaluations of microvilli densities, cross-sections of both duodenum and jejunum were imaged at 12000fold magnification. Counting of microvilli was performed manually in size-defined regions of interest (ROIs) using ImageJ software (Version 1.50a).

Examinations of the diameter of the actin bundle in microvilli were performed on images of cross-sectioned microvilli using ImageJ.

Microvilli length, microvilli diameter and terminal web extension were quantified using TEM images showing the microvilli longitudinally cut (magnification 7000fold; 2–4 measurements each per image). Terminal web extension measurements were done perpendicular to the plasma membrane and in at least about 700 nm distance to adherens junctions.

### Immunolabelling of ultrathin sections

Mice were perfused with PBS and 2% (w/v) PFA in PBS and the gut was dissected. Pieces of intestine (1 mm^3^) were fixed with 0.25% (v/v) glutaraldehyde and 2% (w/v) PFA in 0.1 M sodium cacodylate buffer (pH 7.2) for 3 h and washed thrice with 0.1 M sodium cacodylate buffer. Embedding and ultrathin sectioning was performed as described above.

The sections were incubated for 5 min in TBS (10 mM Tris/HCl pH 7.5, 150 mM NaCl) and subsequently for 30 min in quenching buffer (0.5 M glycine, 10 mM Tris/HCl pH 7.5, 150 mM NaCl). The samples were then washed twice for 1 min with TBS and blocked with labelling buffer (10 mM Tris/HCl pH 7.5 containing 0.5% (w/v) BSA, 0.5% (w/v) gelatine from cold water fish skin and 150 mM NaCl) for 30 min. The sections were incubated with the primary antibody anti-Cobl^DBY^ in labelling buffer over night at 4 °C. After five washing steps with TBS for 5 min each, incubation with secondary gold-conjugated goat anti-guinea pig antibodies in labelling buffer was then performed for 2 h at RT. Following five washing steps with labelling buffer, the sections were washed once with TBS and fixed with 0.1% (v/v) glutaraldehyde in TBS for 2 min. After washing with TBS and ddH_2_O, the samples were stained with lead citrate and analysed by TEM as described above.

### Quantitative analyses of anti-Cobl immunolabelling in the intestine

Blinded images from immunolabelled ultrathin sections of the duodenum were used to quantify the specificity and the subcellular distribution of anti-Cobl immunolabellings in enterocytes. Controls included anti-Cobl immunolabelling attempts on *Cobl* KO intestine sections and WT intestine sections incubated solely with gold-conjugated secondary antibodies.

Determined were the overall immunogold labelling densities and the labelling densities in cytosolic areas including organelle areas but excluding the terminal web and the microvilli, which were evaluated separately.

The cortical area of enterocytes was defined as a 150 nm wide area composed of the 50 apical nanometres of the terminal web (reflecting the maximal extension of immunoconjugates estimated to reach 30 nm plus an extra of 20 nm) and the microvilli base area extending 100 nm from an extrapolated plasma membrane line (connecting the most indented areas of the plasma membrane between the microvilli) towards the microvilli. This generous extension into the microvilli base area was chosen because microvilli bases are sometimes widened and should therefore not yet be considered as nanodomains of microvillar character but rather as part of the cell cortex. Note that the cortical area thereby overlaps with parts of both the terminal web area and the microvilli area.

All areas were defined using ImageJ and gold particles were counted manually using blinded images.

### Intestine pathology examinations

Mouse intestinal samples were embedded as described above for TEM. Sections (thickness, 0.7 µm) were stained with Richardson solution (0.25% (w/v) periodic acid; 0.25% (w/v) Azur II; 0.5% (w/v) methylene blue; 2% (w/v) Na_2_B_4_O_7_) for 4 min and then washed two times with ddH_2_O. Intestines were analysed with a Zeiss Observer Z.1 (with 10x and 40x objectives) and AxioVision 4.8.2 software (Carl Zeiss AG).

### Deep etching and cryo-scanning EM

Pieces of mouse duodenum (approx. 1 mm^3^) were positioned on gold carriers BU012 129-T (BAL-TEC AG), covered with 15% (v/v) methanol and rapidly frozen by liquid nitrogen-cooled propane/ethane. The samples were then placed in a VCT 100 cryo transfer system (BAL-TEC), which was continuously cooled with liquid nitrogen. After connection to a MED 020 high-vacuum coating system (BAL-TEC), samples were cut, deep-etched at −95 °C for 5 min and sputter-coated with 2–3 nm gold.

For visualisation, samples were transferred to a scanning electron microscope Leo 1530 Gemini (Carl Zeiss AG) using a VCT 100 cryo transfer system (BAL-TEC). Images were recorded digitally with an InLens SE detector at 5 kV and processed by ImageJ and Adobe Photoshop.

### Quantitative evaluations of deep-etched cryo-scanning EM images

Terminal web extension measurements were done using blinded images of 10000fold to 30000fold magnification. Two positions per image were measured perpendicular to the extrapolated plasma membrane at the microvilli base using ImageJ. The extension of the terminal web was measured from the apical structure of the terminal web/plasma membrane to the basal structure of the terminal web (i.e. included both structures). The extension of the central area of the terminal web (terminal web minus apical and basal structures of the terminal web) was analysed using the same blinded images and positions and then averaged. Rootlet diameters were also determined using the same set of blinded images (2 measurements per image).

For measurements of the thickness of the apical and the basal structures of the terminal web only images of 20000–38550fold magnification were used. The set of images was blinded using the software Antrenamer 2.12. Four measurements were done per image. Measurements of the extension of the apical structure, the central area and the basal structure of the terminal web were done independently from each other and by independent experimenters to consider maximal putative technical errors. All data per condition was averaged after decoding the blinded images. Remarkably, the terminal web extension for WT and *Cobl* KO mice corresponded very well to the sum of the thickness of the apical structure, the central terminal web and the basal structure of the terminal web (WT, 58 + 153 + 72 = 283 nm vs. measured total terminal web, 272 nm; KO, 52 + 205 + 77 = 334 nm vs. measured total terminal web, 338 nm) highlighting the accuracy of the ultrastructure analysis methods and parameters used.

The occurrence of fine filaments in the central terminal web area that connect terminal web elements was analysed using images of 14900–32630fold magnification. The structures were documented by line drawings in Adobe Photoshop and their numbers were counted manually and expressed per µm terminal web segment for each blinded image.

The diameters of these fine filaments were measured at images with a magnification of 30000fold. The range of length of these filaments was also evaluated at five randomly chosen blinded images exhibiting a 30000fold magnification.

### Statistical analysis

All quantitative data shown represent mean ± SEM.

Prism5 and Prism8 software (SCR_002798) (GraphPad Software) were used to test for normal data distribution and statistical analysis.

Statistical significance calculations between WT and *Cobl* KO were either done by unpaired and two-tailed t-tests or by Mann-Whitney tests, if the data was not distributed normally.

Quantitative determinations of anti-Cobl immunogold labellings of intestinal sections were statistically analysed using One-Way-ANOVA + Dunn’s multiple comparison test.

Statistical significances were marked by *P < 0.05; **P < 0.01; ***P < 0.001 and ****P < 0.001 throughout.

## Supplementary information

Supplementary information.

## Data Availability

The datasets generated during and/or analysed during the current study are available from the corresponding author on reasonable request.
