# Supplementary material for: The actin nucleator Cobl organises the terminal web of enterocytes
Source: Sci Rep. 2020 Jul 7;10:11156. doi: 10.1038/s41598-020-66111-9 (PMC7341751; doi:10.1038/s41598-020-66111-9)

# Supplementary Information

# The actin nucleator Cobl organises the terminal web of enterocytes

Anne J. Beer[1#], Jule González Delgado[1#], Frank Steiniger[2], Britta Qualmann[1*], Michael M. Kessels[1*]

[1] Institute of Biochemistry I, Jena University Hospital - Friedrich Schiller University Jena, 07743 Jena, Germany

[2] Centre of Electron Microscopy, Jena University Hospital - Friedrich Schiller University Jena, 07743 Jena, Germany

# contributed equally

* Correspondence

Britta.Qualmann@med.uni-jena.de & Michael.Kessels@med.uni-jena.de

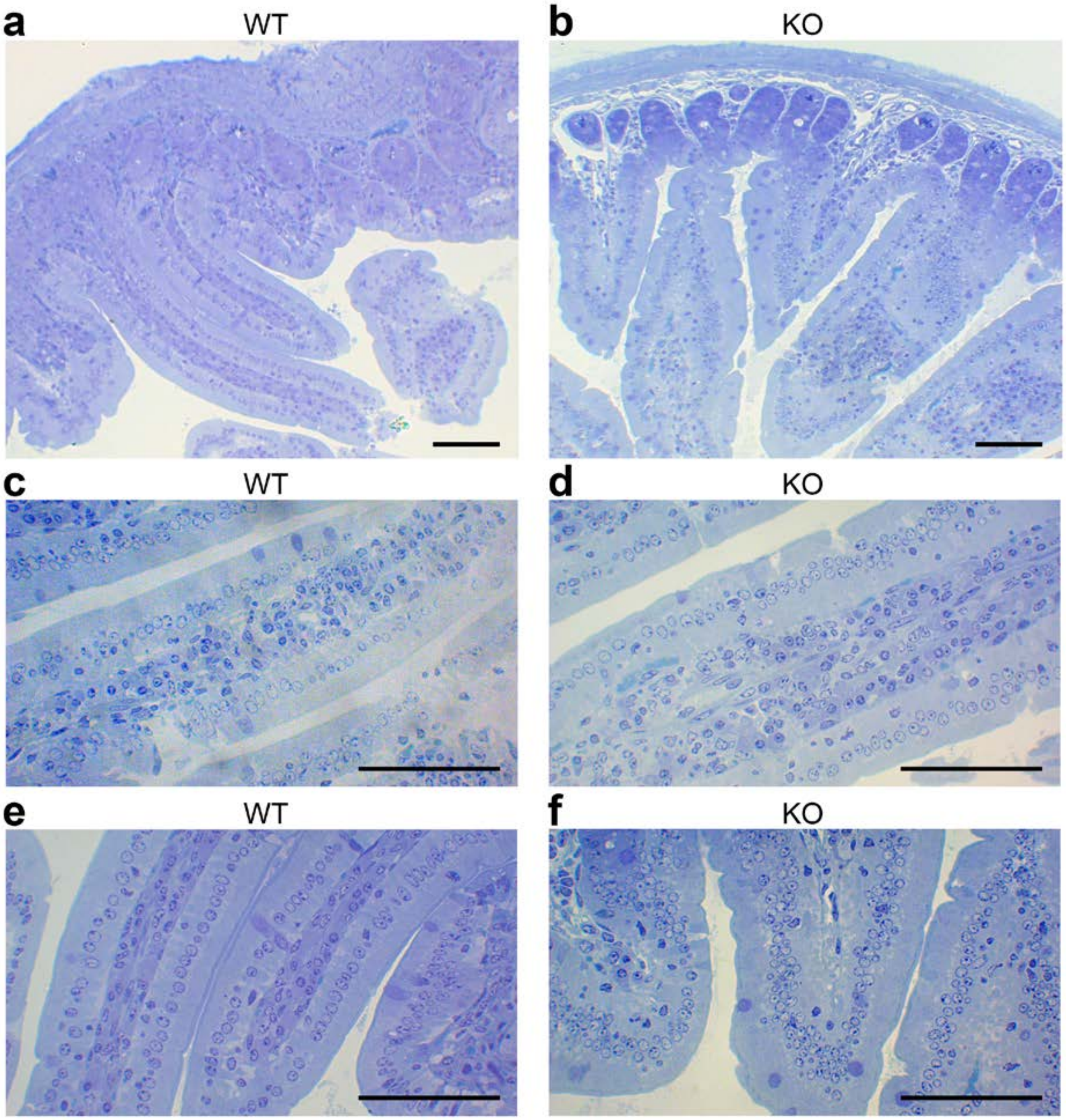


**Supplementary Figure 1. Histological examinations show presence and normal morphology of intestinal villi in *Cobl* KO mice.**

**(a-f)** Bright-field microscopy images of Richardson stainings of sections across the duodenum of WT mice **(a,c,e)** and *Cobl* KO mice **(b,d,f)**. **(c-f)** Details of intestinal villi at higher magnification. Note that no obvious villus defects or other defects of the intestinal tissue can be observed in *Cobl* KO mice by the histological analyses. Bars, 100 µm.

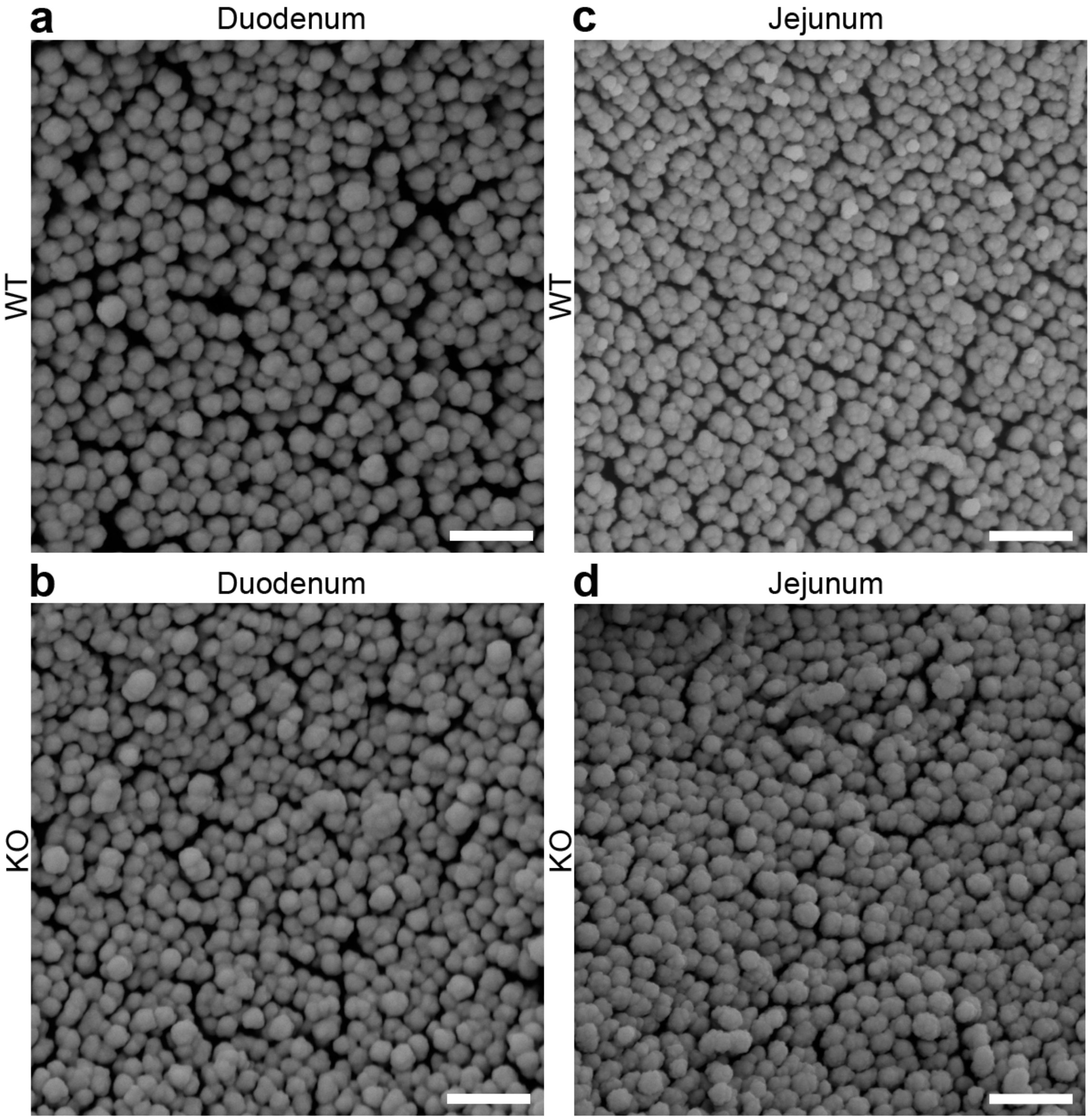


**Supplementary Figure 2. Microvilli densities in both duodenum and jejunum visualised by scanning EM top views onto the intestinal brush borders seem unaffected by *Cobl* KO.**

**(a-d)** Scanning EM images of top views onto the microvilli of duodenum **(a,b)** and jejunum samples **(c,d)** from WT **(a,c)** and *Cobl* KO **(b,d)** mice. Scanning of large intestinal surfaces did neither show lacking microvilli nor irregular microvilli but show regular brush borders with dense microvilli packages. Bars, 400 nm.

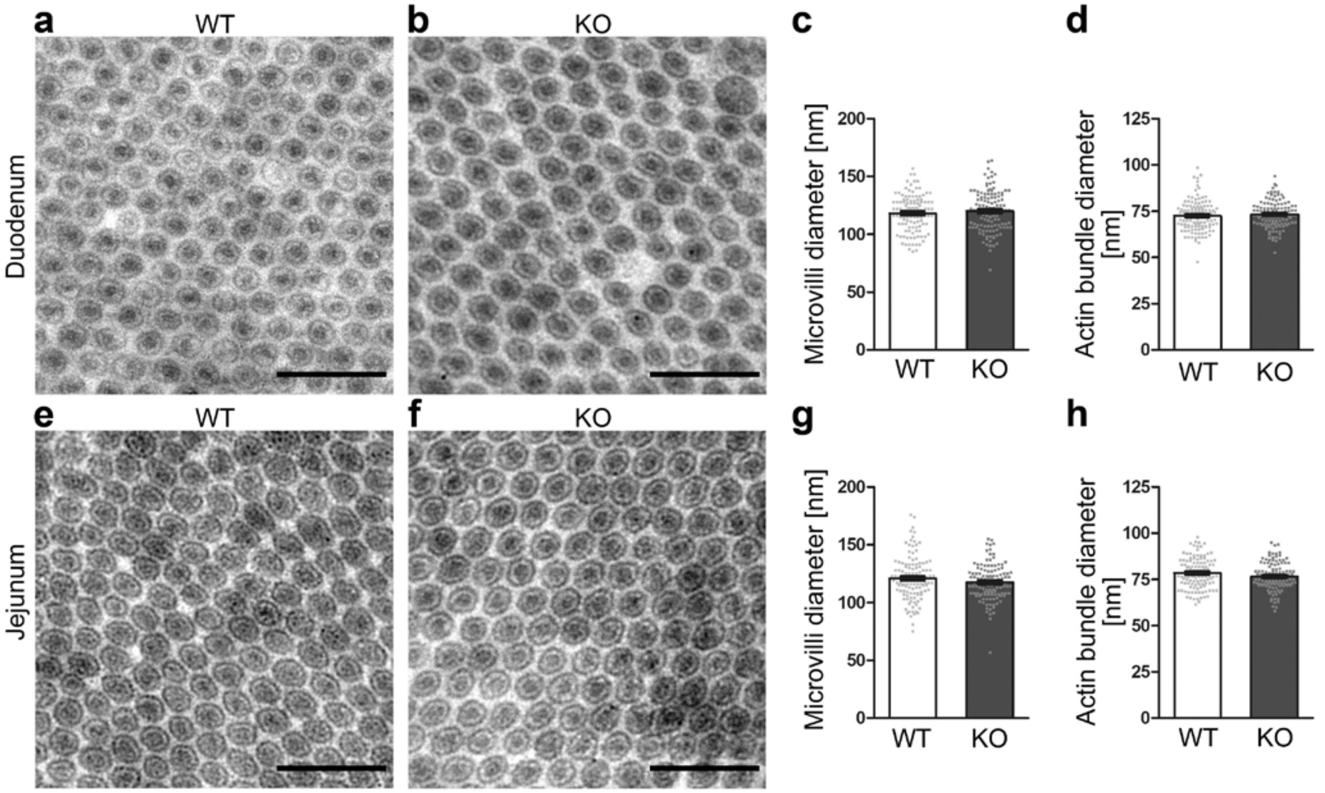


**Supplementary Figure 3. *Cobl* KO mice show normal microvilli and actin bundle diameters in duodenum and jejunum.**

**(a,b,e,f)** Electron micrographs of ultrathin sections of WT **(a,e)** and *Cobl* KO mice **(b,f)**. Images are taken from duodenum **(a,b)** and jejunum **(e,f)**. Bars, 500 nm. **(c,d,g,h)** Blinded, quantitative analyses of microvilli diameter **(c,g)** and actin bundle diameter **(d,h)** from duodenum **(c,d)** and jejunum **(g,h)**. Data, mean±SEM (bar/dot plot overlays). n=120 microvilli and microvillar F-actin bundles from 4 mice/genotype and intestine area each. Statistical analyses, Mann-Whitney **(c)** and unpaired, two-tailed t-test **(d,g,h)**.

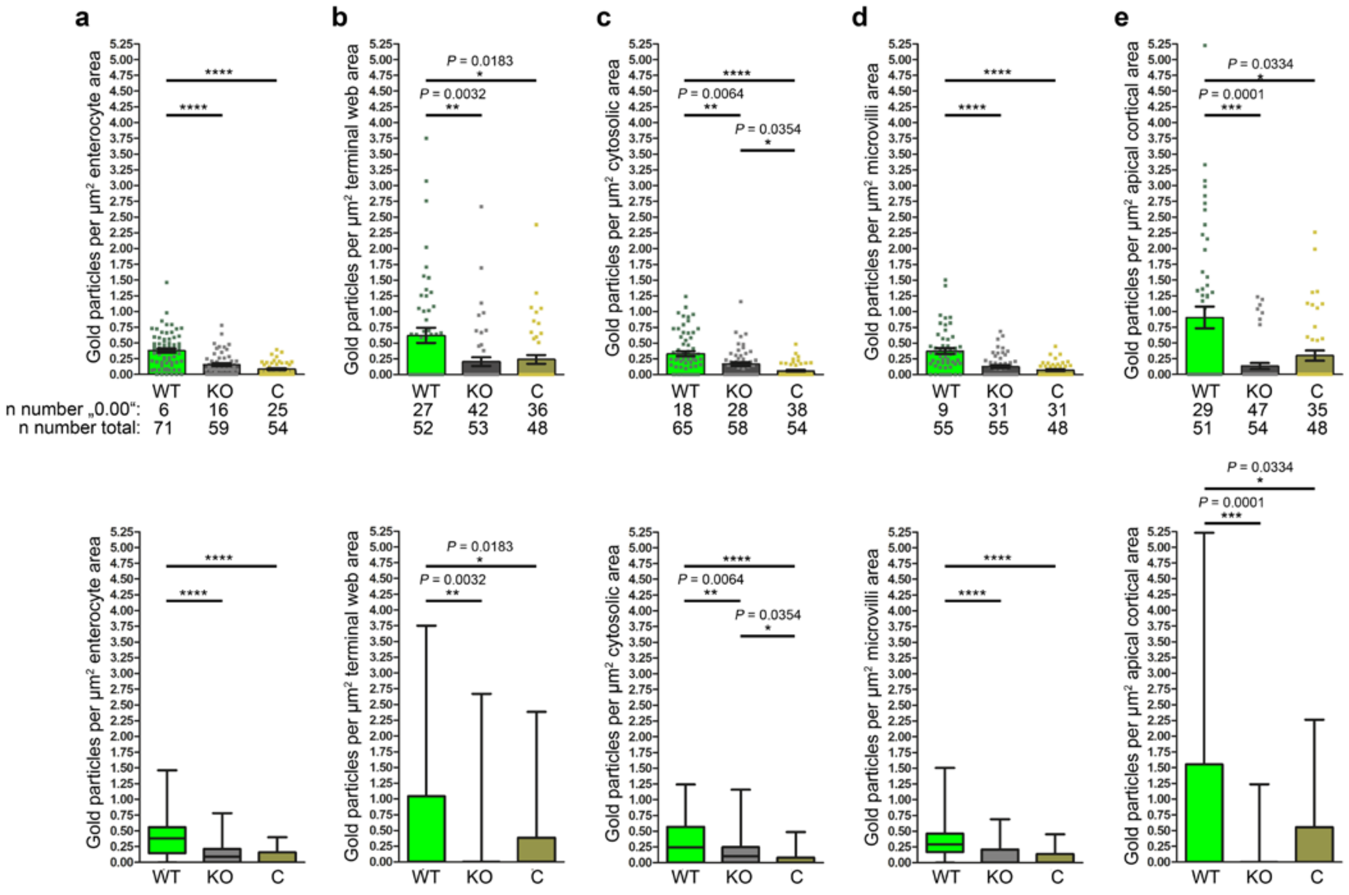


**Supplementary Figure 4. Cobl is primarily located at the cell cortex.**

**(a-e)** Blinded, quantitative analyses of gold particle density in different subcellular areas as shown in Figure 4c-g presented as bar/dot plots, i.e. including all individual data points (upper panels) and as box plots (midline, median; box, 5-95$^{th}$ percentile; whiskers, minimum to maximum values). Note that in order to accommodate higher values, the x-axis is not identical to the bar plots presented in Figure 4c-g but significantly longer in both cases. Note that neither dot plots not box plots are optimal representations of the quantitative data shown in Figure 4. Since subcellular areas, which in the case of in particular the terminal web and the cortex areas are rather small, were analysed, and since labelling densities in immunogold approaches usually are moderate, the labelling density determinations also include zero labelling densities of image areas that were devoid of labelling. These zero data points cannot be fully depicted in the dot plots, as there is not enough space on the zero line. Furthermore,

these data frequently led to a median of zero in the box plots (in this case not visible). Therefore, the numbers of zero-measurements per total n is reported beneath the respective dot plots. The following subcellular areas are shown: enterocyte in general **(a)**, total terminal web area **(b)**, cytosolic areas present in the pictures **(c)**, microvilli **(d)** and cortical area **(e)** from WT and *Cobl* KO mice as well as from WT secondary antibody controls (labelled as “C”). Data, mean±SEM (bar/dot plot overlays). n=48-71 ROIs from 2 mice per each condition (WT, KO, C). One-Way-ANOVA + Dunn’s multiple comparison. *, $P < 0.05$; **, $P < 0.01$; ***, $P < 0.001$; ****, $P < 0.0001$. For $P < 0.0001$, exact $P$ values are not available. Other $P$ values are presented in the figure.

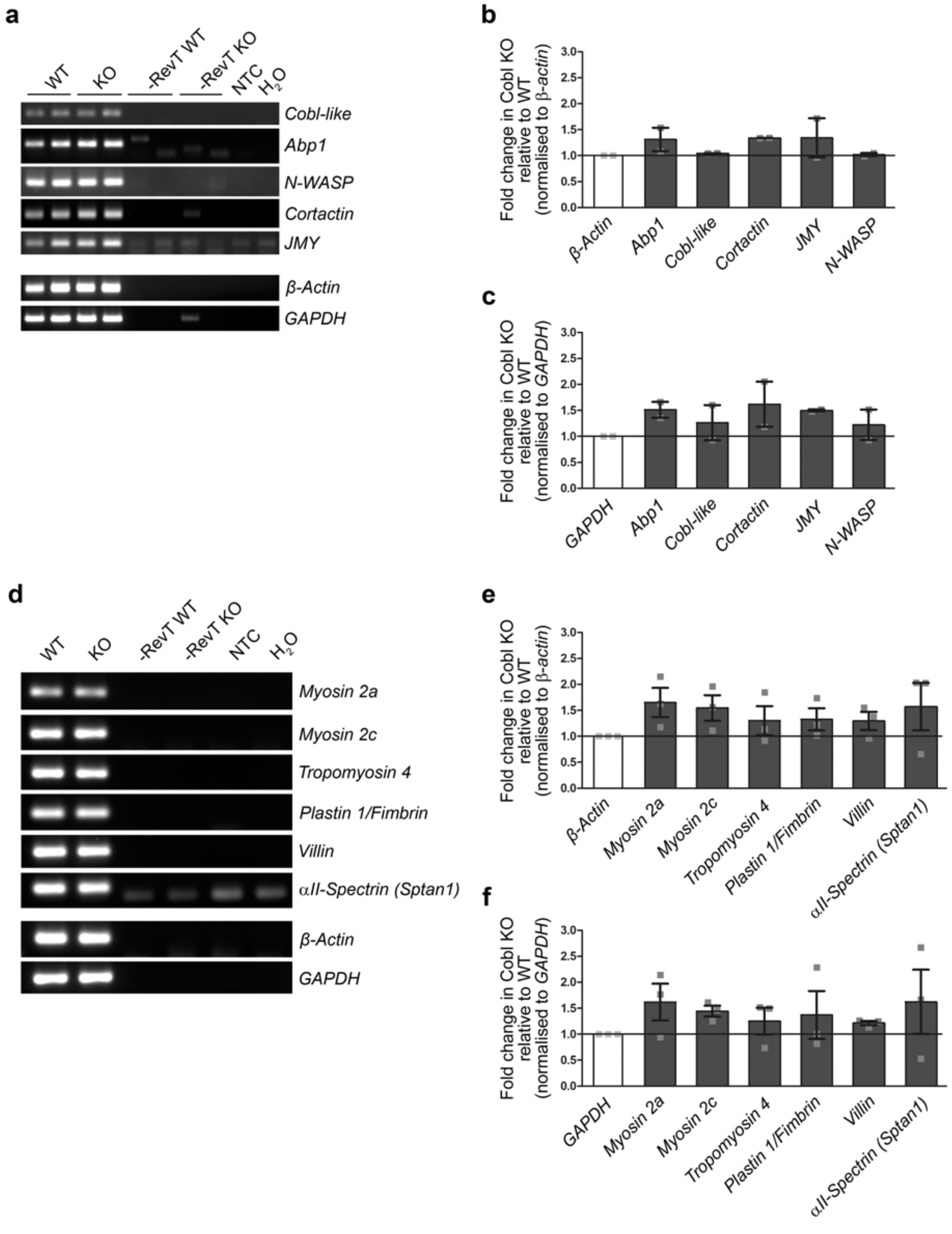


**Supplementary Figure 5. mRNA expressions of *Cobl*-related genes and of further microvillar/terminal web components are not considerably altered in *Cobl* KO duodenum when compared to WT duodenum.**

**(a-c)** qPCR analyses of mRNA levels of cellular components related to Cobl and/or its functions in WT and *Cobl* KO intestinal tissue (duodenum). **(a)** Agarose gel pictures. Negative controls without reverse transcriptase (-RevT), a no-template control (NTC) and water ($H_2O$) were processed and run in parallel. *β-Actin* and *GAPDH* served as controls. **(b,c)** Determination of expression level changes of cellular components related to Cobl and/or its functions (xfold changes per gene in *Cobl* KO relative to WT normalised to either *β-actin* **(b)** or *GAPDH* **(c)** using ΔΔCt analyses (three-fold determination of 2 samples per genotype)). **(d-f)** qPCR analyses of mRNA levels of further cellular components identified in intestinal microvilli and/or the terminal web. Controls as above. **(d)** Agarose gel pictures. **(e,f)** Determination of expression level changes (xfold changes per gene in *Cobl* KO relative to WT normalised to either *β-actin* **(e)** or *GAPDH* **(f)** using ΔΔCt analyses (double determinations of 3 samples per genotype)). Data, mean±SEM (bar/dot plot overlays). Note that neither the tested cellular components related to Cobl and/or its functions **(a-c)** nor the additionally examined microvilli/terminal web components **(d-f)** showed any considerable deviations in mRNA levels in *Cobl* KO duodenum when compared to WT irrespective of whether *β-actin* **(b,e)** or *GAPDH* **(c,f)** was used for normalisation.

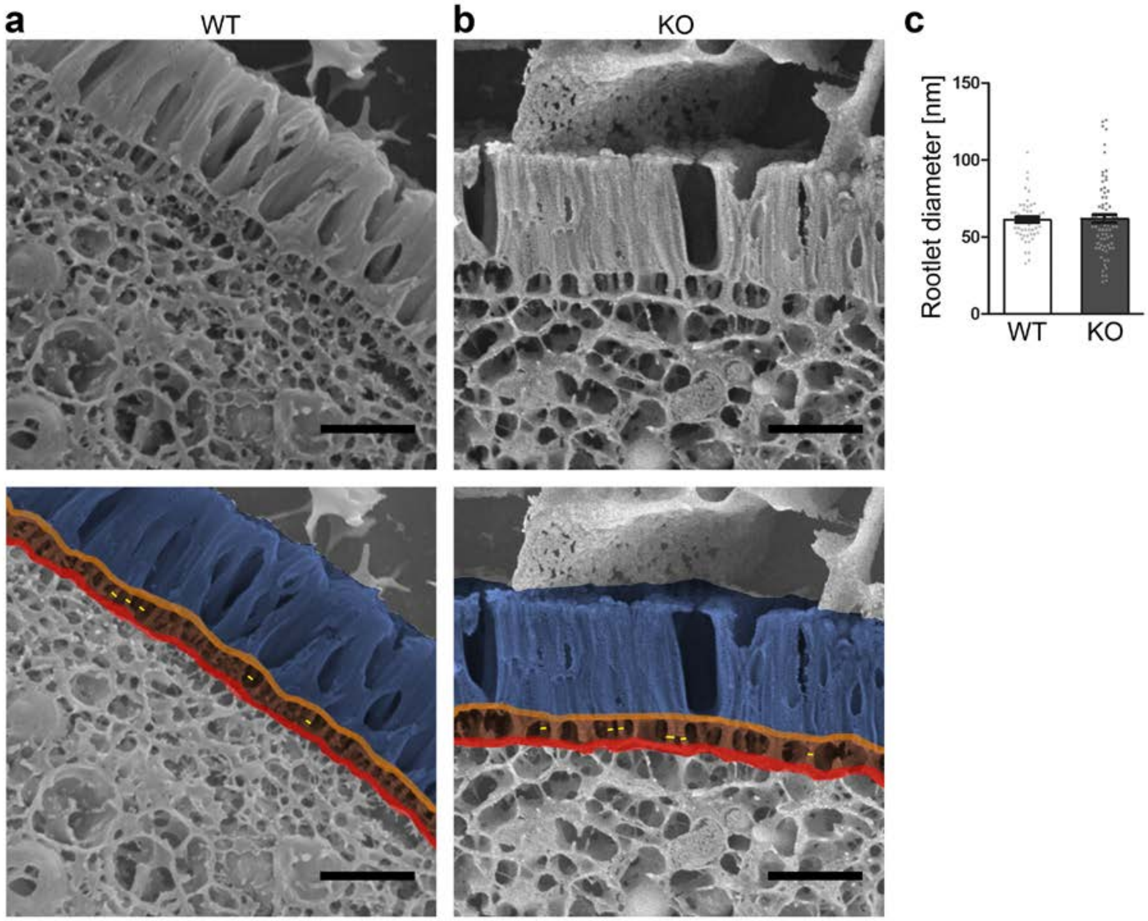


**Supplementary Figure 6. The diameters of the microvillar rootlets in *Cobl* KO mice are unchanged.**

**(a,b)** Cryo-scanning EM images of deep-etched duodenum samples of WT **(a)** and *Cobl* KO mice (**b**) and coloured versions thereof (apical structure of the terminal web in orange, central terminal web in transparent red, basal structure of the terminal web in saturated red and microvilli in blue) with marks visualising rootlet diameter measurements (yellow lines in the lower coloured panels). Bars, 500 nm. **(c)** Blinded, quantitative analyses of rootlet diameters in WT and *Cobl* KO mice. Rootlet diameter **(c)**, n=56 (WT), n=78 (KO) measurements at 28 and 39 images, respectively, from 2 mice/genotype. Data, mean±SEM (bar/dot plot overlays). Statistical analysis, Mann-Whitney test (n.s.).

# Supplementary Data II

**Figure 1a** - **Full blot membranes immunostained**

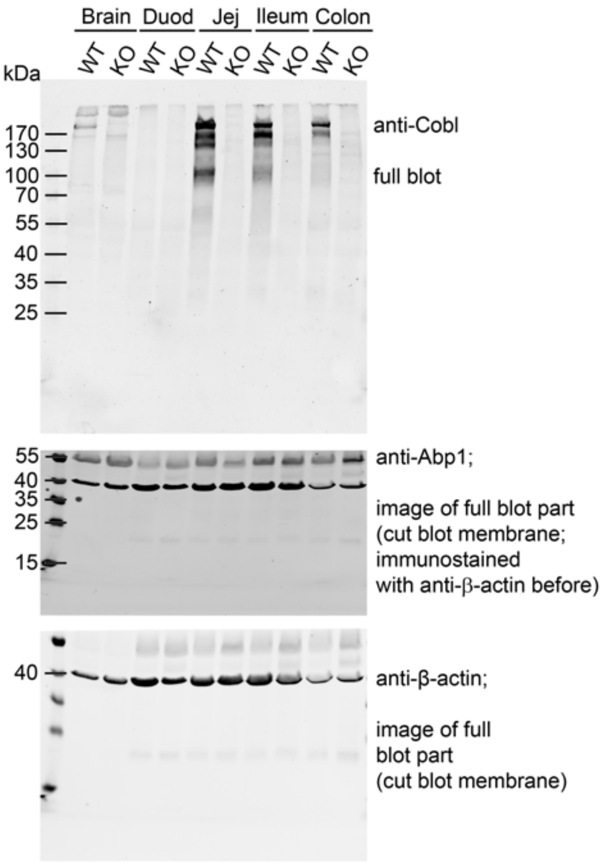

**Figure 1b** - **Full blot membranes immunostained**

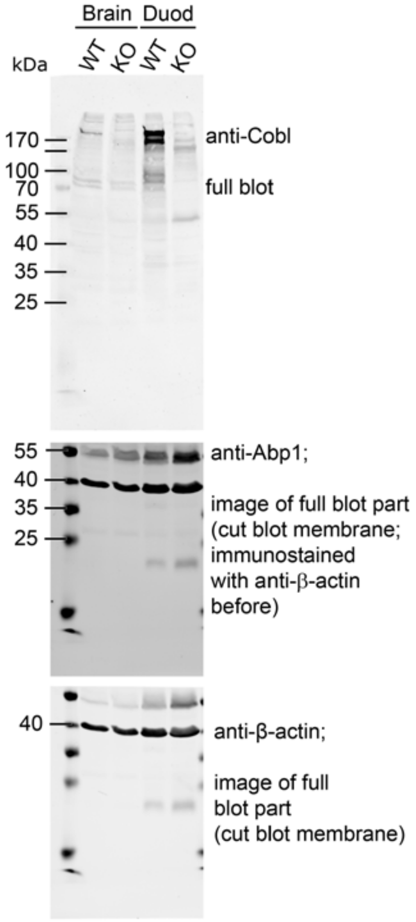

**Figure 1c** - **Full images of agarose gels**

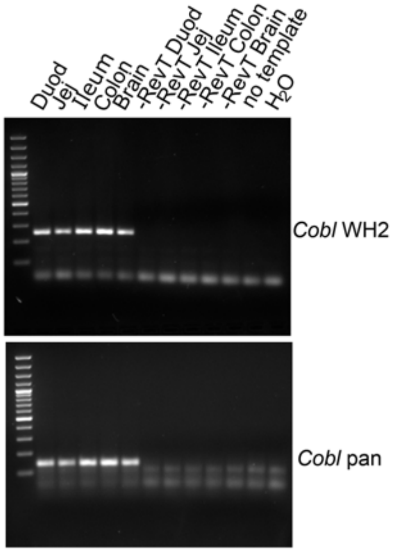

**Figure 1d** - **Full blot membranes immunostained**

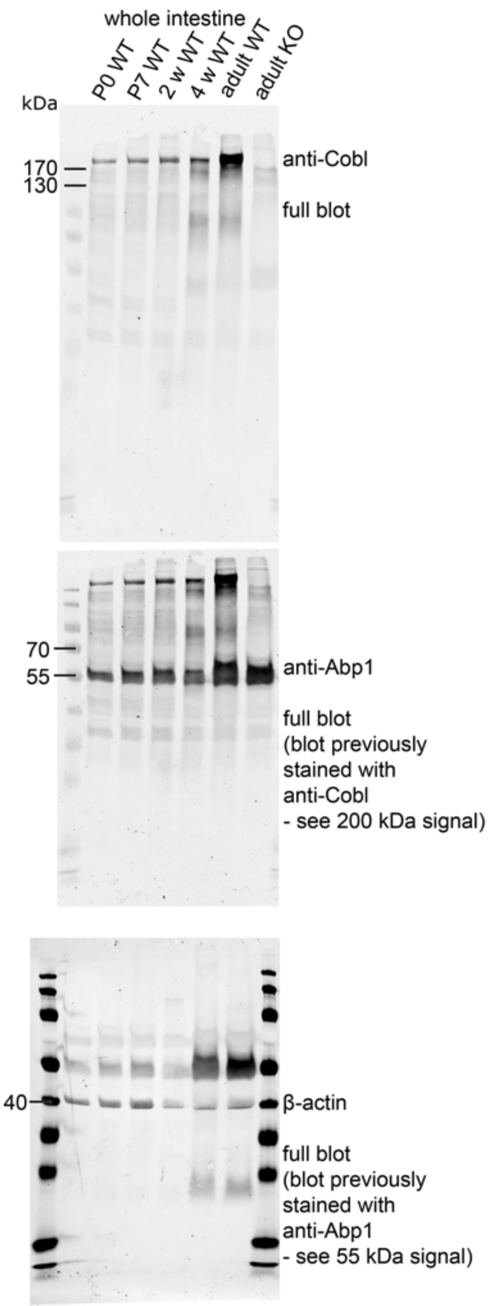

**Figure S5a** - Full images of agarose gels

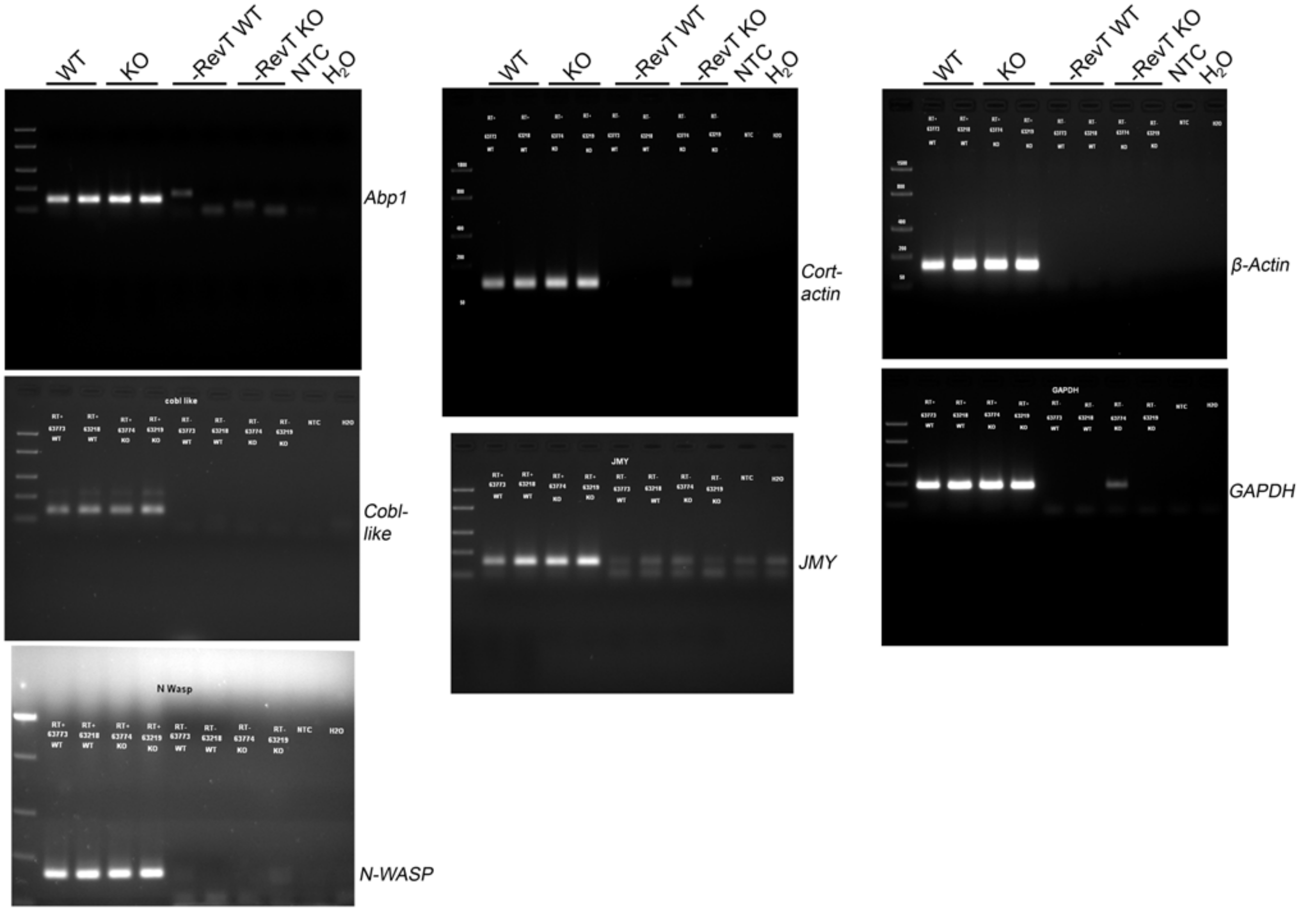

**Figure S5d** - Full images of agarose gels

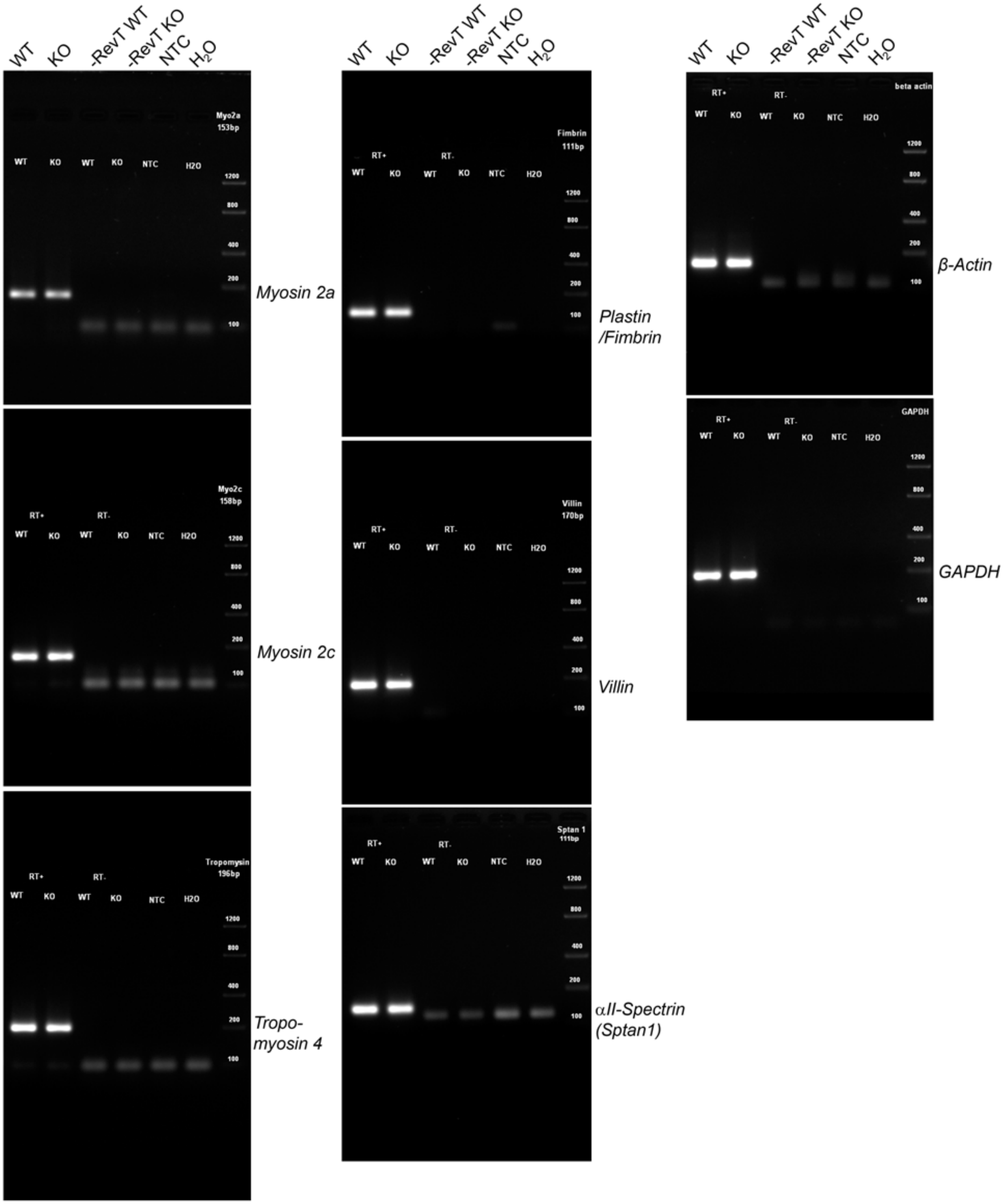

Supplement: Supplementary file 1 — Supplementary information. [file 41598_2020_66111_MOESM1_ESM.pdf]
